# Nerve Growth Factor Pathobiology During the Progression of Alzheimer’s Disease

**DOI:** 10.3389/fnins.2019.00533

**Published:** 2019-07-01

**Authors:** Elliott J. Mufson, Scott E. Counts, Stephen D. Ginsberg, Laura Mahady, Sylvia E. Perez, Stephen M. Massa, Frank M. Longo, Milos D. Ikonomovic

**Affiliations:** ^1^Department of Neurobiology and Neurology, Department of Neurobiology, and Department of Neurological Sciences, Alzheimer’s Disease Laboratory, Barrow Neurological Institute, St. Joseph’s Medical Center, Phoenix, AZ, United States; ^2^Translational Science and Molecular Medicine Michigan State University College of Human Medicine, Grand Rapids, MI, United States; ^3^Center for Dementia Research, Nathan Kline Institute, Orangeburg, NY, United States; ^4^Department of Psychiatry, Department of Neuroscience, and Physiology and NYU Neuroscience Institute, New York University Langone Medical Center, New York, NY, United States; ^5^Department of Neurology, San Francisco VA Health Care System, University of California, San Francisco, San Francisco, CA, United States; ^6^Department of Neurology and Neurological Sciences, Stanford University School of Medicine, Stanford, CA, United States; ^7^Department of Neurology and Department of Psychiatry, Geriatric Research Education and Clinical Center, VA Pittsburgh Healthcare System, University of Pittsburgh, Pittsburgh, PA, United States

**Keywords:** Alzheimer, nerve growth factor, mild cognitive impairment, epigenetics, neurotrophin receptors, biomarker

## Abstract

The current review summarizes the pathobiology of nerve growth factor (NGF) and its cognate receptors during the progression of Alzheimer’s disease (AD). Both transcript and protein data indicate that cholinotrophic neuronal dysfunction is related to an imbalance between TrkA-mediated survival signaling and the NGF precursor (proNGF)/p75^NTR^-mediated pro-apoptotic signaling, which may be related to alteration in the metabolism of NGF. Data indicate a spatiotemporal pattern of degeneration related to the evolution of tau pathology within cholinotrophic neuronal subgroups located within the nucleus basalis of Meynert (nbM). Despite these degenerative events the cholinotrophic system is capable of cellular resilience and/or plasticity during the prodromal and later stages of the disease. In addition to neurotrophin dysfunction, studies indicate alterations in epigenetically regulated proteins occur within cholinotrophic nbM neurons during the progression of AD, suggesting a mechanism that may underlie changes in transcript expression. Findings that increased cerebrospinal fluid levels of proNGF mark the onset of MCI and the transition to AD suggests that this proneurotrophin is a potential disease biomarker. Novel therapeutics to treat NGF dysfunction include NGF gene therapy and the development of small molecule agonists for the cognate prosurvival NGF receptor TrkA and antagonists against the pan-neurotrophin p75^NTR^ death receptor for the treatment of AD.

## Introduction

Alzheimer’s disease (AD) is a progressive and fatal age-associated brain disorder characterized clinically by memory decline, impairment of activities of daily living, neuropsychiatric symptoms, and other behavioral disturbance. Prevalence reports indicate that approximately 18 million people have AD worldwide, with >5.8 million people in the United States (Alzheimer’s Association, 2019). The percentage of cases increases twofold with approximately every 5 years of an increase in age, indicating that 1% of individuals 60 years of age and approximately 30% of people 85 years of age will exhibit the disease. Lacking significant intervention, the number of symptomatic people in the United States will increase to 13.8 million by midcentury (2019). The cost of caring for those with AD will exceed 100 billion United States dollars yearly (Christensen, 2007; Wimo, 2007; Wimo et al., 2017). These alarming statistics stress the overwhelming importance of developing effective treatments for use in the early or prodromal stages of AD.

## Prodromal AD

**FIGURE 1:**
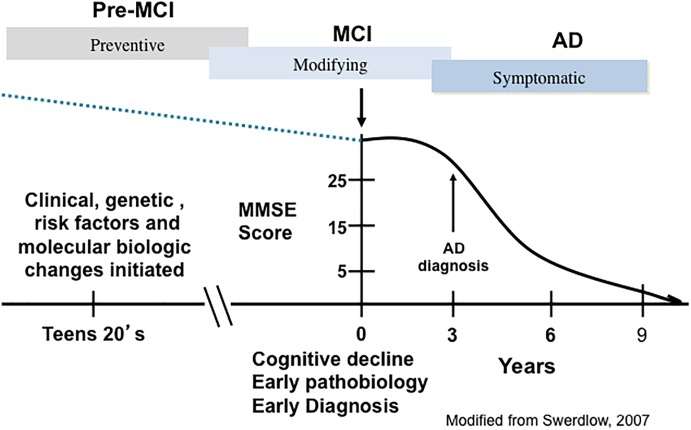
A schematic illustration depicting the trajectory of the clinical and pathological progression of Alzheimer’s disease (AD). PreMCI, pre-mild cognitive impairment; MCI, mild cognitive impairment.

Alzheimer’s disease has an extensive preclinical stage, possibly as early as 15–20 years before the onset of clinical symptoms (Sperling et al., 2014) (Figure 1). Mild cognitive impairment (MCI), a term now synonymous with prodromal AD, is an intermediate phase between normal brain aging and frank dementia when neurofibrillary tangles (NFTs) and amyloid-beta peptide (Aβ) lesions are increased in comparison to those with no cognitive impairment (NCI) (Guillozet et al., 2003; Markesbery et al., 2006; Markesbery, 2010). MCI as a clinical concept was developed from memory clinics, which evaluated milder demented subjects from longitudinal investigations of older cohorts who were tested annually for cognitive status. Such investigations demonstrated that many with earlier, milder cognitive decline failed to show impairment in two cognitive domains as required for an NINDS/ADRDA AD diagnosis (McKhann et al., 1984). These people were defined with an amnestic disorder and termed amnestic MCI (aMCI) (Petersen et al., 1999). Although memory clinics suggested that aMCI was the more common type of MCI leading to AD, it was evident that this entity comprised a minor but a significant aspect of this clinical classification. Overall, the clinical diagnosis of MCI encompasses a heterogeneous population of patients that includes those with isolated memory problems, classified as single domain aMCI, while those with a memory deficit and other cognitive domain impairments are categorized as multi-domain MCI (mdMCI) (Petersen, 2004; Johnson et al., 2010). Amnestic MCI cases are at a greater risk of developing AD (Petersen, 2004; Johnson et al., 2010). A significant proportion of elderly people clinically diagnosed with NCI or with MCI display amyloid plaque and NFT pathology similar to that seen in AD, challenging the pathologically-based concept that these lesions alone hasten dementia onset (Mufson and Kordower, 1999; Price and Morris, 1999; Markesbery, 2010; Mufson et al., 2016a,b).

## Classic AD Lesions

By the twentieth century, neuropathological investigations reported the existence of abnormal extracellular plaques in brains obtained postmortem from older adults with dementia (Blocq and Marinesco, 1892). The German psychiatrist, Dr. Alois Alzheimer, treated Auguste Deter, a 51-year old woman, who presented with signs of paranoia and memory impairment and died 5 years after diagnosis. At autopsy, her brain appeared atrophic, with a loss of neurons, and contained NFTs and senile plaques (SPs). Dr. Emil Kraepelin termed this triad of features “Alzheimer’s disease.” SPs found in the extracellular matrix consist of insoluble fibrils of Aβ, produced from a larger transmembrane amyloid-β precursor protein (APP) by the successive cleavage by the β-site APP cleaving enzyme 1 (BACE1) and the intramembrane γ-secretase complex (Shoji et al., 1992; Thinakaran and Koo, 2008). NFTs consist of intracellular aggregates of hyperphosphorylated tau protein (Trojanowski et al., 1993; Yoshiyama et al., 2013). Although SPs and NFTs are considered the defining pathological hallmarks of AD, Dr. Alzheimer wrote that “…*the plaques are not the cause of senile dementia, but only an accompanying feature of senile involution of the central nervous system”* (Alzheimer, 1911). Despite this statement, the AD research field has been driven by the “amyloid cascade hypothesis” (Hardy and Selkoe, 2002) and treatment strategies continue to revolve around the development of anti-amyloid drugs to remove plaque deposition. However, virtually all anti-amyloid clinical trials have not met their primary end-point, the improvement of cognition (Hampel et al., 2015, 2018). This lack of drug efficacy lends support to the concept that amyloid may be an early biomarker of AD but not necessary for a clinical decline. More likely AD is a multifaceted polygenic disease of which amyloid is a partner in the pathogenesis of this disease. Contrary to the amyloid hypothesis, a large body of literature suggests the loss of cognition involves the selective vulnerability of multiple neurotransmitter pathways leading to a massive cortical disconnection syndrome.

## Cholinotrophic Basal Forebrain Defects During the Progression of AD

**FIGURE 2:**
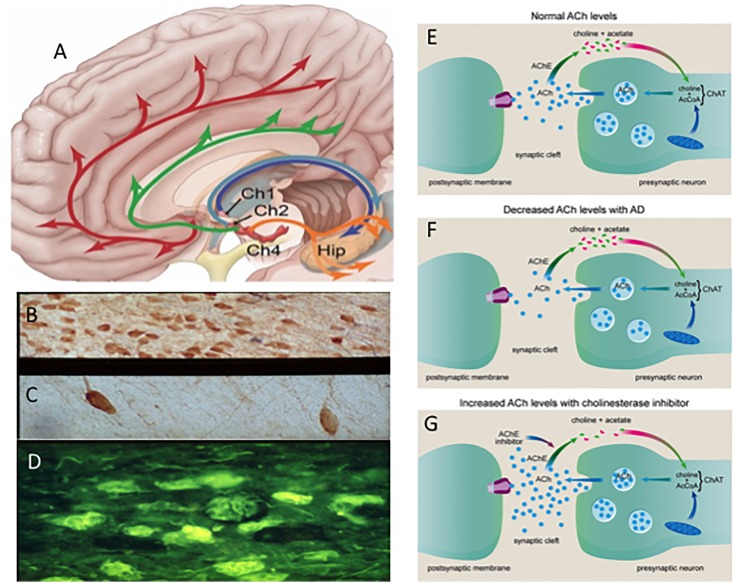
**(A)** Schematic drawing of the cholinotrophic cortical and hippocampal projection systems. Photomicrographs showing images of cholinotrophic neurons in an aged control **(B)**, reduction in AD **(C)** and thioflavin (yellow) tangle-bearing p75^NTR^ neurons (dark blue) **(D)** in AD. Cartoon showing changes in acetylcholine (ACh) between a normal **(E)** and AD **(F)** and the effect of acetylcholinesterase inhibitors at the cholinergic synapse **(G)**. Ch1, medial septal cholinergic cell group; Ch2, vertical limb of the diagonal band cholinergic cell group Ch4; nucleus basalis cholinergic cell group and Hip, hippocampus. Red and green arrows indicate bilateral Ch4 projections; dark blue and light blue arrows indicate septal/diagonal band projections to the hippocampus.

For over 30 years degeneration of cholinergic basal forebrain (CBF) neurons, which innervate the entire neocortex and hippocampus (Figure 2A–D) has been investigated as a key neurotransmitter system affected early in the disease that may be a target for AD treatment (Hampel et al., 2018). The “cholinergic hypothesis” of AD (Bartus et al., 1982) gained momentum with the finding that acetylcholinesterase inhibitors (AChEIs) have significant symptomatic effects in AD patients (Summers et al., 1986) This led to the development of a larger family of acetylcholinesterase inhibitors (AChEIs) (Hampel et al., 2018) (Figure 2E–G), which remains one of the few classes of FDA approved drugs for the treatment of AD (Johannsen, 2006; Mangialasche et al., 2010; Hampel et al., 2018). For example, the AChEI, donepezil, has been shown to reduce basal forebrain atrophy within the nucleus basalis of Meynert (nbM) and the medial septum/diagonal band in prodromal AD, demonstrating a structural effect (Cavedo et al., 2017) as well as symptomatic relief.

Recently, there has been a resurgence of interest in the CBF projection system in the field of early-onset dementia (Douchamps and Mathis, 2017; Hampel et al., 2018). Imaging studies provide evidence of the importance of dysregulated basal forebrain circuitry in signaling related to cognitive decline (Ballinger et al., 2016), dysregulation of the default mode network (DMN) critical for executive function, episodic memory (Nair et al., 2018), and propagation of cortical atrophy early in the evolution of the disease (Schmitz and Nathan Spreng, 2016), and as a pre-symptomatic biomarker for AD (Ho et al., 2008; Grothe et al., 2012). This renewed interest in cholinergic cortical projection neurons to the DMN and other cortical sites during the onset of AD underscores the critical need to understand the mechanistic factors underlying dysfunction of this projection system.

## Nerve Growth Factor During the Progression of AD

**FIGURE 3:**
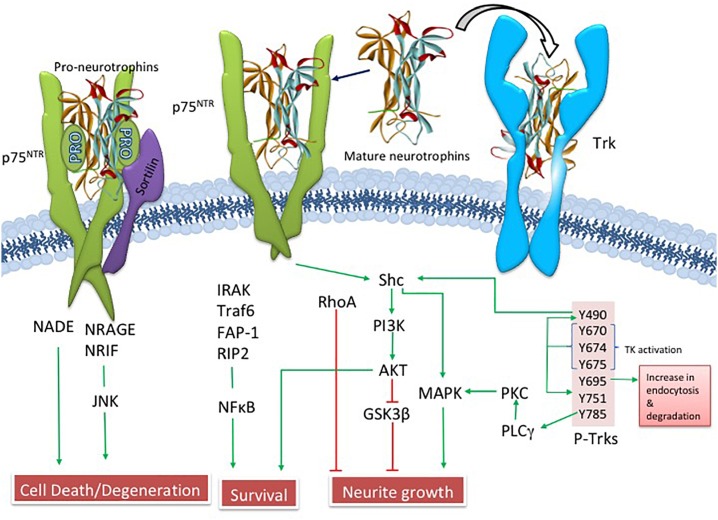
Illustration showing NGF related upstream and downstream pathways.

Since Ramon y Cajal suggested that brain cells require “special food,” researchers have searched for growth-stimulating agents that play a role in neuronal survival (Henry, 1998). Levi-Montalcini and Cohen (Levi-Montalcini, 2000), received the Nobel Prize for their discoveries showing that the trophic substance nerve growth factor (NGF) underlies the selective survival of cultured neurons. They were the first to suggest the neurotrophic hypothesis of neuronal survival. NGF is a product of a single gene found on chromosome 1, which gives rise to a 27 kiloDalton (kDa) and a 35 kDa proNGF precursor protein (Francke et al., 1983; Edwards et al., 1988), which are proteolytically cleaved to a mature biologically active peptide (Edwards et al., 1988; Lee et al., 2001). ProNGF, not mature NGF, is the primary form found in the human brain (Fahnestock et al., 2001). NGF binds to its cognate tropomyosin-related kinase A (TrkA) receptor and the p75 pan-neurotrophin receptor (p75^NTR^) (Ibanez, 2002; Chao, 2003; Kaplan and Miller, 2004). NGF binding to TrkA activates downstream survival pathways by activating Akt (Ulrich et al., 1998) while proNGF and p75^NTR^, together with its co-receptors sortilin (Nykjaer et al., 2004) and neurotrophin receptor homolog-2 (NRH2) (Murray et al., 2004) activate the c-Jun N-terminal protein kinase (JNK) related to cellular apoptosis (Nykjaer et al., 2005) (Figure 3). Clinical trials have shown that NGF has therapeutic potential to enhance CBF survival and neuroplasticity in AD (Tuszynski et al., 1990; Tuszynski and Blesch, 2004; Tuszynski et al., 2015).

## NGF and the Progression of AD

**FIGURE 4:**
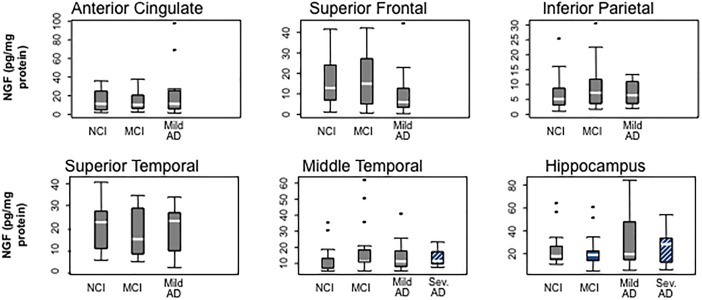
Box plots showing the stability of NGF protein levels with the cortex and hippocampus during the progression of AD. Reproduced from Mufson et al. (2003).

Although for many years it was hypothesized that cholinotrophic basal forebrain cortical and hippocampal projection neurons degenerate due to loss of NGF in AD (Hefti and Mash, 1989; Tuszynski et al., 1990; Smith et al., 1999), studies reported unchanged (Goedert et al., 1989; Allen et al., 1991; Murase et al., 1993; Jette et al., 1994), decreased (Hellweg et al., 1998) or increased (Crutcher et al., 1993; Scott et al., 1995; Fahnestock et al., 1996; Narisawa-Saito et al., 1996; Hellweg et al., 1998; Hock et al., 2000) NGF levels using tissue from severe AD subjects. However, NGF levels were preserved in five cortical regions (superior frontal, superior temporal, middle temporal, anterior cingulate, and inferior parietal cortex) and hippocampus in people who came to autopsy with a clinical diagnosis of MCI, mild AD, and severe AD (Figure 4) (Mufson et al., 2003). In contrast, others report an increase in cortical and hippocampal *Ngf* mRNA and protein in end-stage AD, where volume loss could lead to increased concentrations of NGF per weight or volume (Crutcher et al., 1993; Jette et al., 1994; Scott et al., 1995; Fahnestock et al., 1996; Narisawa-Saito et al., 1996; Hellweg et al., 1998; Hock et al., 2000) or the translation from *Ngf* to encoded NGF protein may be compromised or expression levels differ between AD cases. We reported a wide range of NGF activity in a cohort ranging from early to late-onset AD cases and some of the highest and lowest levels of NGF were seen in end-stage AD cases (Scott et al., 1995), suggesting that within a given cohort, NGF levels can be differentially affected by age at disease onset or differences in disease process. In this study, there was no relationship between cortical choline acetyltransferase (ChAT) activity, the rate-limiting enzyme for acetylcholine synthesis, and levels of NGF, nor between reduced numbers of ChAT- (Gilmor et al., 1999), TrkA- (Mufson et al., 2000), or p75^NTR−^ (Mufson et al., 2002b) containing neurons in MCI and mild AD. Moreover, the lack of a correlation between the apolipoprotein ε4 genotype and NGF levels is interesting, since ApoE ε3 and ε4 alleles are reported to be associated with a greater decrease in cholinergic markers in end-stage AD (Poirier et al., 1995). Although over 90% of the severe AD cases we examined from the Rush Religious Orders Study (RROS) contained at least one ApoE ε4 allele, NGF levels did not differ across the clinical groups evaluated (Mufson et al., 2003). These observations suggest that ApoE ε4 genotype does not directly affect the metabolism of NGF.

## Expression of NGF Receptors During the Progression of AD

Cholinergic basal forebrain neuron function is dependent upon the binding of NGF to its cognate receptor TrkA, as well as its pan-neurotrophin p75^NTR^, which lends support to the suggestion that dysregulation of NGF and its receptors underlie cholinergic neuron dysfunction in AD. TrkA receptors and p75^NTR^ are produced within the perikarya of CBF neurons and anterogradely transported to the cortex and hippocampus the sties of NGF production (Schwab et al., 1979). Within CBF neurons, mature NGF binds to the TrkA receptor, activating signal transduction pathways that regulate neuronal survival induced by NGF (Kaplan and Miller, 2004). However, p75^NTR^ is a positive modulator of NGF/TrkA binding (Kaplan and Miller, 2004), and exhibits several context-dependent functions including the stimulation of apoptotic or cell death pathways (Bamji et al., 1998; Yoon et al., 1998; Frade, 2000; Friedman, 2000; Lee et al., 2001; Roux and Barker, 2002). In this regard, the specific downstream effects of p75^NTR^ are dependent upon its interaction with various receptor chaperones (Mamidipudi and Wooten, 2002; Nykjaer et al., 2004; Teng and Hempstead, 2004).

**FIGURE 5:**
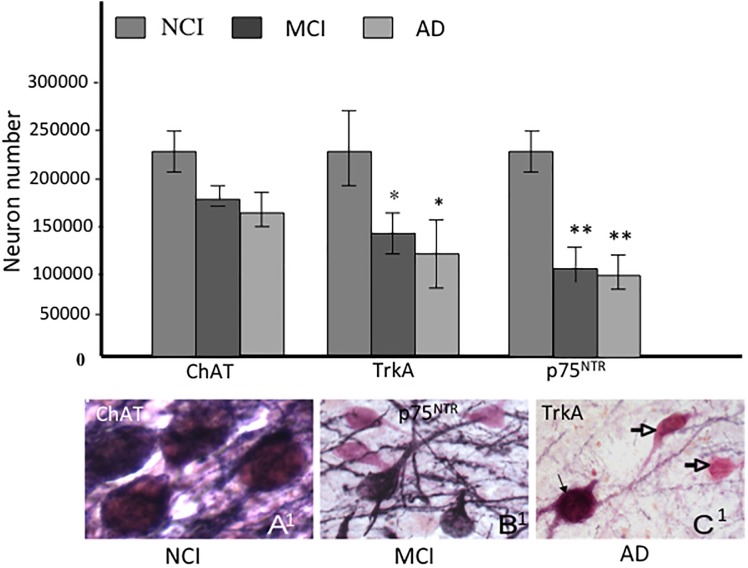
Histograms showing the differential reduction in cholinergic, TrkA and p75^NTR^ immunoreactive neurons during the progression of AD. A^1^- C^1^. Photomicrographs of dual labeled nucleus basalis neurons showing phenotypic downregulation of p75^NTR^ (dark blue) compared to ChAT (pink) positive neurons between no cognitive impairment (NCI), mild cognitive impairment (MCI) and AD. Note the loss of p75^NTR^ immunoreactive staining of ChAT-positive cells in MCI and AD (open arrows). Black arrow indicates dual stained neuron in AD (C^1^), ^∗^ and ^∗∗^ indicate *p* < 0.05 and 0.01, respectively.

In order to evaluate whether the number of CBF neurons containing NGF receptors is altered early in the progression of AD, we examined tissue from RROS subjects clinically categorized as NCI, MCI, or AD (Gilmor et al., 1999). Interestingly, the numbers of ChAT-containing neurons were stable in MCI and mild AD, while TrkA- and p75^NTR^-immunoreactive neurons were significantly decreased compared to NCI, indicating a phenotypic downregulation of receptors supporting CBF function rather than frank neuronal degeneration in MCI (Gilmor et al., 1999) (Figure 5). The phenotypic loss of cholinotrophic markers due to atrophy, rather than overt cholinergic cell loss, is consistent with animal model studies of septal cholinergic neuron axotomy via fimbria-fornix transection and excitotoxicity (Hefti, 1986; Williams et al., 1986; Ginsberg and Martin, 1998). In AD, reduced cortical TrkA levels positively correlated with lower cognitive performance as assessed by the Mini-Mental State Exam (MMSE) (Counts et al., 2004), suggesting that decreased nbM and cortical NGF receptor protein levels may mark the early onset of AD.

## Cortical proNGF Levels During the Progression of AD

Alterations in proNGF, the NGF precursor protein, has received extensive study in clinical pathological investigation of components of the cortical DMN, which includes frontal cortex, posterior cingulate, precuneus, superior temporal cortex (Perez et al., 2015), and the hippocampus (Mufson et al., 2012b), which contribute to cognitive dysfunction during the progression of AD (Sperling et al., 2014). ProNGF isolated from AD cortex induces apoptosis in neuronal cell cultures by interacting with p75^NTR^ via a mechanism dependent upon γ-secretase shedding of the receptor, whereas proNGF isolated from control brain does not activate apoptosis (Pedraza et al., 2005). ProNGF levels are increased in the lateral parietal cortex of patients who died with a clinical diagnosis of MCI or mild AD compared to those with NCI (Peng et al., 2004). In contrast, precuneus proNGF levels were stable until end-stage AD (Perez et al., 2015), similar to that of the frontal cortex (Fahnestock et al., 2001, 2004; Podlesniy et al., 2006) and hippocampus (Al-Shawi et al., 2008; Mufson et al., 2012b), all of which suggest alterations of proNGF in the diseased brain. Western blotting found no changes in the levels of TrkA, p75^NTR^ and the co-receptor, sortilin within the precuneus (Perez et al., 2015), and hippocampus (Mufson et al., 2010) across clinical groups. ProNGF binds with a higher affinity to p75^NTR^, which is enhanced in the presence of sortilin to induce apoptosis (Lee et al., 2001; Nykjaer et al., 2004; Pedraza et al., 2005; Al-Shawi et al., 2008). Homeostatic regulation of NGF receptors, combined with the binding of proNGF to TrkA (albeit with less affinity than mature NGF), results in the activation of downstream pathways involved in CBF neuron function (Fahnestock et al., 2001, 2004) as well as the induction of neurotrophic activity via the binding with less affinity to the TrkA receptor (Fahnestock et al., 2001, 2004). The finding that p75^NTR^ levels remain stable in the precuneus and other cortical regions (Counts et al., 2004; Mufson et al., 2012b) during the onset of AD may be related to the demonstration of a *de novo* appearance of p75^NTR^ cortical neurons in AD (Mufson and Kordower, 1992).

**FIGURE 6:**
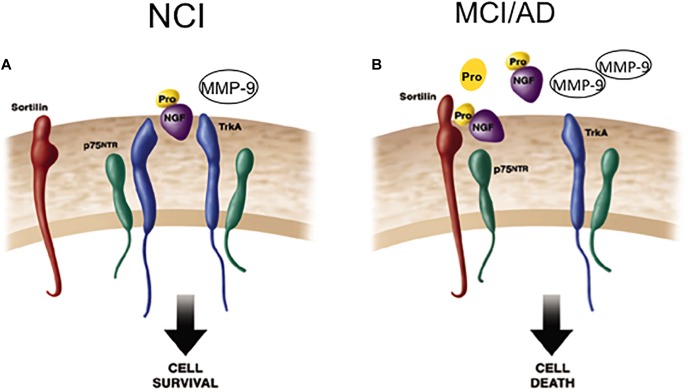
Cartoon demonstrating the shift from cell survival to cell death related to NGF activity during the progression of AD. **(A)** ProNGF/TrkA complex activates cell survival mechanisms, which is facilitated by the co-expression of p75^NTR^ on the cell surface in the healthily aged brain. ProNGF also creates a signaling complex by simultaneously binding to sortilin a co-receptor for proNGF. **(B)** In AD, elevated cortical proNGF in the face of reduced TrkA enhances binding of proNGF to p75^NTR^ /sortilin complex. Since sortilin acts as a molecular switch governing a p75^NTR^ mediated pro-apoptotic signal, increased proNGF triggers cell death in the face of decreased TrkA. Modified from Mufson et al. (2012a).

The pro-apoptotic effect(s) of p75^NTR^-mediated proNGF signaling is dependent on interactions with p75^NTR^ and sortilin, a Vps10p domain trafficking protein that acts as a cell surface co-receptor with p75^NTR^ to mediate proNGF-activated cell death. This family of receptors is gaining importance, due to its potential involvement in AD (Nyborg et al., 2006). Sortilin activates p75^NTR^-induced apoptosis following proNGF treatment (Nykjaer et al., 2004), suggesting a role in cell death (Mamidipudi and Wooten, 2002; Roux and Barker, 2002). Blocking this binding event precludes binding of proNGF to p75^NTR^ and subsequent cell degeneration (Bronfman and Fainzilber, 2004; Kaplan and Miller, 2004; Nykjaer et al., 2004; Teng et al., 2005). It is possible that p75^NTR^ signaling in response to proneurotrophins depends upon the identity and efficacy of the bound co-receptor. Notably, cortical levels of sortilin remain stable, similar to p75^NTR^ during the progression of AD. Perhaps pro-survival or pro-apoptotic signaling in CBF neurons is dependent upon changes in the stoichiometry of TrkA, p75^NTR^, the availability of select co-receptors, and the physiological role of proNGF within different milieus during the early stage of AD. Shifting the balance of these factors may change the response that proNGF binding activates within CBF neurons during the progression of AD (Figure 6). Defining these interactions will be key to the development of neurotrophic strategies for dementia (Bruno et al., 2004; Longo et al., 2007). If proNGF binds p75^NTR^ *in vivo* and induces apoptosis (Lee et al., 2001; Nykjaer et al., 2004), it will be crucial to develop drugs that block proNGF binding to p75^NTR^. In contrast, if proNGF binds with TrkA to induce cell survival (Fahnestock et al., 2004), then the development of drugs that enhance this interaction could provide neuroprotection in AD.

## NGF Metabolic Pathways During the Progression of AD

**FIGURE 7:**
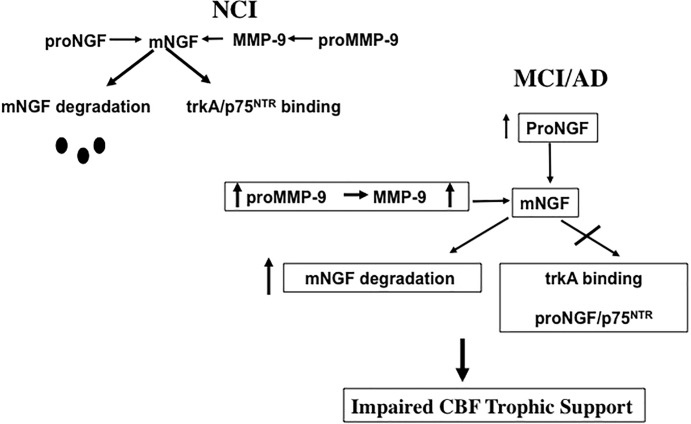
Diagram illustrating the effect of an increase of proMMP-9/MMP-9 has upon the ability of NGF to support cholinotrophic basal forebrain neurons during the progression of AD.

Defects in the metabolic pathways regulating the maturation and degradation of the NGF/proNGF complex may play a key role in CBF dysfunction. Recently, a protease cascade, which converts proNGF to mature mNGF and degrades mNGF in the extracellular space by the coordinated activity of plasminogen, tissue plasminogen activator (tPA), neuroserpin, matrix metalloproteinase 9 (MMP-9) and tissue inhibitor of matrix metalloproteinase 1 (TIMP-1) was shown to be defective in AD (Bruno and Cuello, 2006). In this regard, the upregulation of MMP-9 protein levels and activity were reported in the frontal and parietal cortex in MCI and AD, which was inversely associated with cognitive performance (Bruno et al., 2007), and may drive changes in NGF/proNGF activity (Figure 7). We suggest that increased proMMP-9 and MMP-9 compromises NGF support of CBF neurons during the transition from NCI to MCI (Figure 7). Interestingly, a similar increase in cortical proNGF (Iulita et al., 2014) and reduction in TrkA-positive CBF neurons (Sendera et al., 2000) has been reported in Down syndrome (DS), suggesting an overlap in NGF neurotrophic dysregulation in these disorders. Both AD and DS cases display cortical SP and NFT pathology and develop dementia by midlife (Mann and Esiri, 1989), further connecting these neurological conditions. It has been suggested that levels of metalloproteinases (MMPs) in blood, urine, and cerebrospinal fluid (CSF) may act as potential biomarkers for AD (Zucker et al., 1999; Lorenzl et al., 2003, 2008). It is of interest to examine whether or not MMPs are dysregulated in the precuneus, allowing for the stable metabolic NGF/proNGF complex regulation early in AD. Notably, proNGF levels were not associated with increased soluble Aβ_1–42_ or fibrillar Aβ [3H] Pittsburgh Compound B (PiB) binding, but instead with compact/cored 6-CN-PiB– positive plaques in AD (Perez et al., 2015), suggesting that fibrillar deposits of Aβ, rather than its soluble forms, may play a role in the upregulation of proNGF we found in the precuneus. Neurodegeneration is a consequence of Aβ_1–40_ binding to p75^NTR^ (Knowles et al., 2009) and CBF perikarya when Aβ oligomers are delivered to the brains of wild type but not p75^NTR^ deficient mice (Simmons et al., 2014). Interestingly, CBF degeneration was halted following the depletion of the neurotrophin-binding domain of p75^NTR^ in a mouse model of AD (Knowles et al., 2009). These findings suggest that p75^NTR^ signaling is involved in Aβ-induced degeneration and implicate it as an AD therapeutic target.

## Hippocampal proNGF and Downstream Pathways During the Progression of AD

**FIGURE 8:**
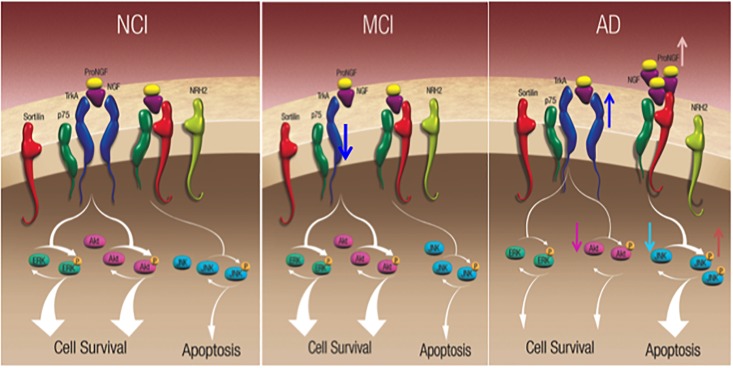
Schematic diagrams showing alteration in hippocampal neurotrophic protein levels during the progression of AD. Reproduced from Mufson et al. (2015).

The hippocampus is a part of the mesial temporal lobe memory circuit. It develops extensive NFTs but lesser amyloid pathology in the early stages of AD (Hyman et al., 1984, 1990; Braak and Braak, 1991; Arriagada et al., 1992) and receives a major cholinergic input from the medial septal and vertical limb of the diagonal band neurons (Mesulam et al., 1983). Since these septohippocampal cholinergic projection neurons are also dependent upon NGF and its cognate receptors for their survival and degenerate in AD, studies were performed to determine alterations in the hippocampal NGF/proNGF system. Western blot analysis revealed a significant increase in hippocampal proNGF levels in AD but not MCI (Mufson et al., 2012b) in contrast to the neocortex (Bruno et al., 2009; Perez et al., 2015). Of interest is the observation of a significant reduction in TrkA protein levels in MCI hippocampus compared to NCI and AD and a return to NCI levels during the transition from MCI to AD (Mufson et al., 2012b) (Figure 8). The decrease in TrkA in the face of stable proNGF early in AD may enhance proNGF/p75^NTR^/sortilin/NRH2 binding, ultimately shifting the balance from pro-survival to pro-apoptotic signaling in the hippocampus (Figure 8). The upregulation of hippocampal TrkA levels is yet another example of human brain resilience (Mufson et al., 2016a,b) to slow disease progression.

Nerve growth factor and proNGF activate numerous downstream cell survival and apoptotic signaling pathways, respectively (Figure 8). The cell survival protein Erk, which is activated by TrkA phosphorylation, activates nuclear effectors involved in gene transcription (Zhu et al., 2001). Precuneus (Perez et al., 2015) and hippocampal (Mufson et al., 2012b) levels of total Erk, phospho-Erk, and phospho-Erk/Erk ratio are unchanged between NCI, MCI, and AD. In contrast, stress-activated kinase phospho-JNK, and the ratio of phospho-JNK to JNK were significantly increased in the AD precuneus (Figure 8) (Perez et al., 2015) and hippocampus (Mufson et al., 2012b), whereas total JNK levels were stable similar to the AD hippocampus (Mufson et al., 2012b). Bcl2 a component of the JNK signaling pathway involved in the activation of apoptotic enzymes was upregulated in the precuneus in AD but not MCI (Perez et al., 2015). Of particular interest was the finding that phospho-JNK and the density of AT8 tau-positive NFTs and neuropil threads (NTs) are positively related during the onset of AD, supporting the observation that JNK activation mediates tau phosphorylation at Ser202/Thr205 (AT8 site) (Goedert et al., 1997; Reynolds et al., 1997). Therefore, the activation of JNK pro-apoptotic signaling may play a role in episodic memory impairment in AD.

## Cholinotrophic Basal Forebrain Neuron Gene Expression During AD Progression

**FIGURE 9:**
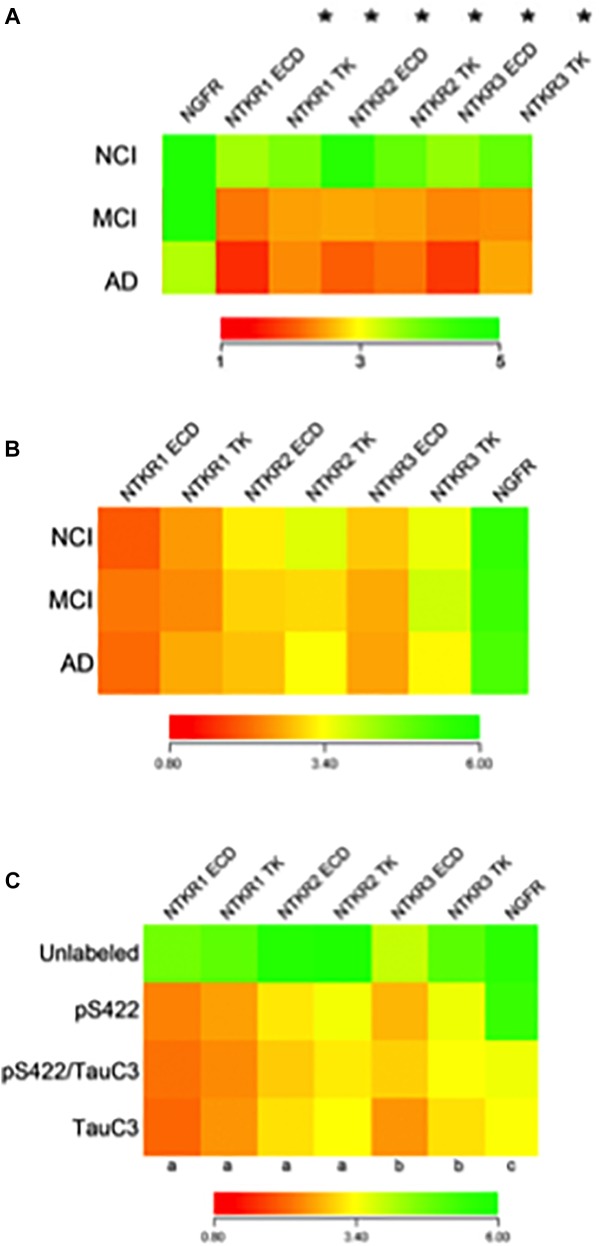
Findings derived from single cholinotrophic neuron profiling during the progression of AD. **(A)** Heatmap demonstrating downregulation of *Ntrk1* (TrkA), *Ntrk2* (TrkB), and *Ntrk3* (TrkC) {both the extracellular domain (ECD) and the tyrosine kinase domain (TK)} (asterisk), but not *Ngfr* (p75^NTR^) during the progression of dementia. Trk gene expression in MCI is intermediate to AD, indicating a step-down effect from NCI to MCI to AD. **(B)** Heatmap of the relative expression profiles for select neurotrophin transcripts in pS422+ nbM neurons. No differences in Trk receptors or *Ngfr* were found in MCI or AD compared to NCI. **(C)** Downregulation of Trk receptors was found in pS422+ nbM neurons compared to unlabeled control neurons. Downregulation of *Ngfr* was associated with the neoepitope TauC3. Key: (a) unlabeled > pS422, *p* < 0.001; (b) unlabeled > pS422, *p* < 0.01; (c) pS422 > pS422+/TauC3+, *p* < 0.01. **(A)** Adapted with permission from Ginsberg et al. (2006b). **(B,C)** Adapted with permission from Tiernan et al. (2018a).

The identification of the genetic signature of ‘selectively vulnerable’ CBF neurons compared to relatively spared neurons during the onset of AD is crucial for the development of transcriptionally aided drug design to target therapeutics to intervene with the onset of AD. A transcriptionally-driven therapeutic approach may be more likely to preserve brain connectomes including the NGF dependent CBF system that plays a key role in the pathogenesis and onset of dementia, especially during early stages of the disease process (Mufson et al., 2012a). Studies comparing gene expression profiles of CBF neurons identified by p75^NTR^ (Mufson et al., 1989) display a dysregulation of select synaptic-related markers (e.g., downregulation of synaptophysin and synaptotagmin 1 among others), protein phosphatases/kinases (e.g., downregulation of protein phosphatases 1 and 2 subunits and upregulation of cyclin-dependent kinase 5) along with endosomal-lysosomal markers (e.g., upregulation of lysosomal markers cathepsin D, rab4, rab5, and rab7) in MCI and AD compared to age-matched NCI subjects (Ginsberg et al., 2006a,b, 2010; Counts et al., 2011). Moreover, significant downregulation of *TrkA*, *TrkB*, and *TrkC* was seen in single CBF neurons microaspirated from the nbM of MCI and AD compared to NCI (Ginsberg et al., 2006b) (Figure 9A), consistent with observations in another vulnerable cell type, hippocampal CA1 pyramidal neurons (Ginsberg et al., 2000, 2010). These findings revealed an intermediate reduction in MCI with the greatest decrement in AD compared to NCI. Moreover, expressed sequence tagged cDNAs (ESTs) [i.e., ESTs targeted to both the extracellular domain (ECD) and tyrosine kinase (TK) domains of Trk receptors] were downregulated. A ‘step down’ dysregulation of Trk expression, may in part, underlie CBF neuron demise associated with the clinical presentation of AD. Supporting this concept is the finding that downregulation of *TrkA* was associated with several measures of cognitive decline, including the MMSE, a composite global cognitive score (GCS), Episodic, Semantic, Working Memory, Perceptual Speed, and Visuospatial domains as well as Braak NFT stage and neuritic plaque (NP) load within the basal forebrain and hippocampus (Ginsberg et al., 2006b, 2019). Hence, *Trk* gene expression defects may provide a molecular marker for the transition from MCI to frank AD (Ginsberg et al., 2019). In contrast, p75^NTR^ transcript levels were stable in CBF neurons across the clinical diagnostic groups (Ginsberg et al., 2006b), which was an intriguing finding compared to the significant reduction of p75^NTR^-immunopositive nbM perikarya in MCI and AD compared to NCI (Gilmor et al., 1999). The discrepancy between p75^NTR^ protein and transcript expression in CBF neurons suggests a disconnection between mRNA transcription and protein translation during disease onset. CBF single population observations in postmortem human brain tissues suggest a relative selectivity in the alteration of the family of cognate NGF receptors during the progression of AD, and that neurotrophic deficits precede or occur during the earliest stages of cognitive decline and neuropathology.

## Cholinotrophic Neuron TAU Pathology During the Progression of AD

Coincident with altered neurotrophic factor dysfunction during disease progression, CBF neurons also develop intracellular tau inclusions that appear as globose NFTs as well as NTs in MCI and AD (Sassin et al., 2000; Mesulam et al., 2004; Wu et al., 2005; Vana et al., 2011). The human brain contains three isoforms of tau with three tandem repeats (3Rtau; *Mapt1*, *Mapt3*, and *Mapt5*) and three tau isoforms with four tandem repeats (4Rtau; *Mapt2*, *Mapt4*, and *Mapt6*). Custom-designed microarray evaluation of CBF neurons did not reveal changes in any of the six tau transcripts between AD, MCI and NCI subjects (Ginsberg et al., 2006a). However, a significant shift in the ratio of 3Rtau/4Rtau ratio was observed with a decrease in 3Rtau expression relative to 4Rtau levels for all tau transcripts. Tau transcript data suggest a fluctuation in gene dosage for 3Rtau and 4Rtau within CBF neurons in MCI and AD, which was not seen in normal aging (Ginsberg et al., 2006a).

Single neuron expression profiling investigations have addressed the extent to which levels of transcripts encoding neurotrophin receptors are altered in individual nbM neurons labeled for the pretangle marker pS422+, the late stage caspase-cleaved tau marker TauC3+ or pS422/TauC3+ compared to unlabeled neurons obtained from NCI, MCI, and AD cases provided by the RROS (Tiernan et al., 2018a). Quantitative analyses compared transcript signal intensities between clinical stages or between tau neuronal phenotypes. Comparison of transcript expression in pS422+ nbM neurons microaspirated from each clinical stage revealed no statistical differences (Figure 9B). However, when analyzed independent of clinical diagnosis, expression levels of key genes regulating neurotrophin receptor expression were altered as classified by a phenotypic transition from unlabeled to pS422+ to pS422+/TauC3+ to TauC3+ in nbM neurons (Figure 9C). Compared to unlabeled, pS422+ nbM neurons showed a significant downregulation of six mRNAs encoding the intracellular TK and extracellular ECD domains of the neurotrophin receptors *TrkA* (*Ntrk1* TK, 50% downregulation; *Ntrk1* ECD, 53%), *TrkB* (*Ntrk2* TK, 45%; *Ntrk2* ECD, 42%), and *TrkC* (*Ntrk3* TK, 38%; *Ntrk3* ECD, 35%) (Figure 9C). In addition, we found that these same transcripts are significantly downregulated in neurons containing the early pretangle tau antibody Tau Oligomeric Complex 1 (TOC1) (Counts, unpublished observations), lending support to the hypothesis that neurotrophic dysfunction occurs before frank NFT formation.

In contrast, transcript levels of the mRNA encoding the pan-neurotrophin receptor p75^NTR^ (*Ngfr*) were not decreased until the appearance of TauC3. This expression data complements our stereologic finding demonstrating that TauC3 and p75^NTR^ did not co-localize within the CBF at the protein level (Mufson et al., 2002b; Vana et al., 2011). Prior studies reported that Trk receptors expression levels are downregulated in nbM neurons in MCI and AD relative to NCI (Ginsberg et al., 2006b). On the other hand, it was found that *Ntrk* transcripts were downregulated by the phenotypic transition from nbM non-labeled to pS422+ neurons, whereas no difference was found in *Ntrk* expression in pS422+ neurons from NCI, MCI, and AD cases (Figure 9C).

We previously performed antibody imunostaining for TOC1 (Patterson et al., 2011; Ward et al., 2013) and p75^NTR^ to quantify pretangle tau oligomeric assemblies, which most likely are the more neurotoxic species of tau (Berger et al., 2007; Maeda et al., 2007; Kopeikina et al., 2011; Lasagna-Reeves et al., 2012; Sahara et al., 2013) within CBF neurons during the progression of AD (Tiernan et al., 2018b). Here the number of p75^NTR^+/TOC1+ nbM neurons progressively increased from NCI to MCI to AD, whereas single TOC1+ nbM neurons were lower in NCI and MCI but increased in AD. A sub-analysis of p75^NTR^+, p75^NTR^+/TOC1+, and TOC1+ nbM neurons in NCI cases with a low Braak score (Stages I–II) compared to a high Braak score (Stages III–V) revealed a significant increase in the number of p75^NTR^+/TOC1+ dual-immunolabeled neurons in NCI-high pathology compared to NCI-low pathology cases. The reduction of p75^NTR^+ nbM neurons was associated with poorer GCS and MMSE performance test scores. TOC1 primarily co-localized with pS422 in NCI, but the transition to MCI and AD was marked by a shift from TOC1+/pS422+ toward triple-labeled TOC1+/pS422+/MN423+ neurons, implying a specific, linear order of epitope occurrence in nbM cholinotrophic neurons during disease progression. This arrangement suggests that aberrant phosphorylation primes the tau protein toward additional phosphorylation and conformational events (Luna-Munoz et al., 2007; Bertrand et al., 2010) that facilitates oligomerization (Iqbal et al., 2013). Given the findings that prefibrillar tau pathology is related with molecular and cellular alterations within nbM neurons (Tiernan et al., 2016, 2018b), the appearance of prefibrillar oligomeric tau likely precedes cell loss. Evidence implicating tau pathology as a driver of neurotrophic dysfunction is seen in tau transgenic mice and tau-transfected neuronotypic cells, which display a downregulation of the trophic substance brain-derived neurotrophic factor (BDNF) (Rosa et al., 2016). On the other hand, NGF regulates tau turnover (Sadot et al., 1996) and post-translational modifications including phosphorylation, cleavage, and ubiquitination (Nuydens et al., 1997; Shelton and Johnson, 2001; Babu et al., 2005; Amadoro et al., 2011), suggesting that neurotrophic abnormalities initiate tau pathology within CBF neurons (Canu et al., 2017). Moreover, the reduced microtubule-binding capacity of tau and/or the somatodendritic accumulation of tau may contribute to axonal degeneration and associated NGF/TrkA signaling dysregulation (Mufson et al., 2002a; Schindowski et al., 2008). Additionally, proNGF induces tau hyperphosphorylation *in vitro* via the enhanced activity of GSK3β (Shen et al., 2018). Based on these collective results future analysis of the potential interactions between aberrant tau metabolism and disrupted neurotrophic signaling in cholinotrophic nbM neurons (Capsoni et al., 2000).

## Cholinotropic Epigenetic Alterations During the Progression of AD

Histone acetylation and deacetylation are also involved in CBF neuron function via their regulation of ChAT (Aizawa and Yamamuro, 2010; Aizawa et al., 2012; Bekdash, 2016) indicating a potential role for epigenetics in neuronal selective vulnerability in AD. Evidence is growing that histone deacetylases (HDACs), epigenetic enzymes with deacetylase activity, located within the nucleus and cytoplasm of neurons play a role in AD pathogenesis (Ding et al., 2008; Guan et al., 2009; Xu et al., 2011; Cook et al., 2012; Graff et al., 2012). Several HDACs are related with cellular events dysfunctional in AD, including endoplasmic reticulum stress (HDAC4) (Shen et al., 2016), autophagy (HDAC6) (Pandey et al., 2007), mitochondrial transport (HDAC6) (Chen et al., 2010), tau hyperphosphorylation (HDAC6) (Ding et al., 2008) and Aβ and tau accumulation (SIRT1) (Julien et al., 2009; Lalla and Donmez, 2013). However, whether epigenetic dysregulation occurs in cholinotrophic nbM neurons remains under-investigated in AD.

**FIGURE 10:**
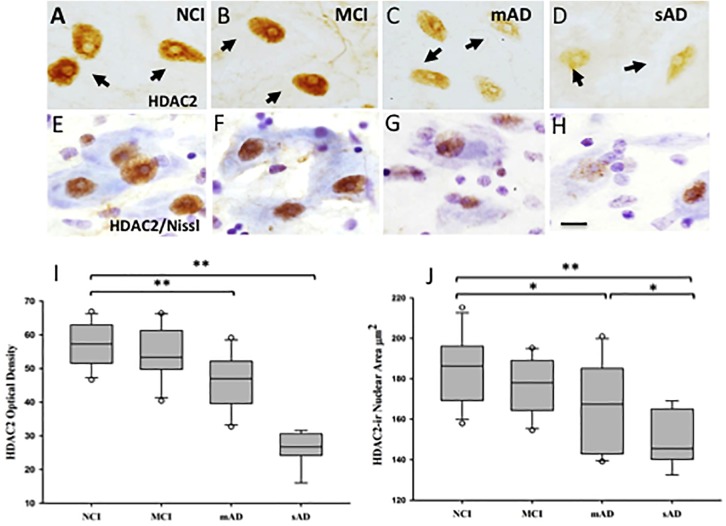
Photomicrographs of single HDAC2 **(A–D)** and HDAC2 and cresyl violet dual stained **(E–H)** nucleus basalis neurons. Nuclei positive for HDAC2 lost their rounded shape and were displaced to the periphery of the soma in the MCI, mAD, and sAD groups. **(I)** Box plots showing HDAC2-ir was significantly decreased in mAD compared to NCI and MCI (*p* < 0.001), and sAD compared to NCI, MCI, and mAD (*p* <0.001). **(J)** Box plots showing area of HDAC2-ir nuclei was significantly decreased in mAD compared to NCI (*p* = 0.03) and in sAD compared to NCI (*p* < 0.001), MCI (*p* < 0.001), and mAD (*p* = 0.04) groups. Circles in box plots indicate outliers. ^∗^*p* < 0.05; ^∗∗^*p* < 0.001. Scale bar: 10 μm. Reproduced from Mahady et al. (2019).

Of the HDACs, HDAC2 has received extensive investigation due to a role in the modulation of transcripts involved in cognition via chromatin plasticity regulation (Dawson and Kouzarides, 2012; Graff and Tsai, 2013; Volmar and Claes, 2015). In this regard, a clinicopathological investigation revealed alterations in HDAC2-immunoreactive (ir) nuclei within nbM neurons during the onset of AD (Mahady et al., 2019). This study revealed that normally rounded HDAC2-ir nbM nuclei appeared ovoid, flattened, and eccentrically located within the soma in MCI, mild AD (mAD) and severe AD (sAD) (Figure 10A–H). HDAC2 nuclear intensity was significantly reduced in sAD compared to the other clinical groups examined (Figure 10D,H). Moreover, HDAC2-ir nuclear intensity was significantly reduced in mAD compared to the NCI and MCI groups (Figure 10I). This study further demonstrated that HDAC2 nbM nuclear intensity was not significantly different in NCI and MCI. The sAD nuclear area was significantly smaller than observed in NCI, MCI, and mAD (Figure 10J). In mAD, HDAC2-ir nuclei displayed significantly smaller area compared to NCI. HDAC2-ir nuclear intensity was found to correlate with working memory and a GCS (Mahady et al., 2019). A decline in the number of nbM p75^NTR^ immunoreactive neurons decreased across disease stages and was related to a reduction in HDAC2 nuclear immunoreactivity (Mahady et al., 2019). Similarly, a reduction in HDAC2 nuclear immunoreactivity was inversely related to an increase in the number of AT8 pretangle-bearing nbM neurons. Quantitation of the intensity of HDAC2 nuclei revealed a significant reduction in non-tangle bearing p75^NTR^-positive neurons in mAD and sAD compared to NCI and MCI. NbM neurons triple-labeled for p75^NTR^, the pretangle maker AT8 or late-stage tau epitope, TauC3, displayed an even larger decrease in HDAC2 immunoreactivity in AD compared to non-tangle bearing p75^NTR^ neurons at each disease stage (Mahady et al., 2019). Within-group analysis indicated HDAC2-ir was highest in non-tangle bearing cholinergic perikarya in each clinical group (Mahady et al., 2019). Interestingly, HDAC2 nuclear immunoreactivity was further decreased in HDAC2/AT8/Thioflavin-S or HDAC2/TauC3/Thioflavin-S neurons in MCI and mAD. These findings suggest that a reduction in HDAC2 expression occurs before the onset of fibrillar tau pathology and that this alteration is exacerbated by phosphorylated and conformational tau epitopes in nbM neurons during the progression of AD. Although ChAT mRNA expression and protein levels are epigenetically modulated by hyperacetylation of the core promoter region of the *ChaT* transcript (Aizawa et al., 2012), decreased HDAC2 nuclear levels were found within cholinergic nbM neurons in MCI, but significantly decreased ChAT nbM protein levels were seen only in AD (Mahady et al., 2019). These phenomena suggest that HDAC2 nuclear protein downregulation does not alter neuronal ChAT activity early in the disease process. Immunohistochemical analysis of HDACs in cortical cholinergic nbM projection sites demonstrated differential regional findings. For instance, although HDAC1 and HDAC2 are reduced in AD entorhinal cortex (Mastroeni et al., 2010), HDAC2, but not HDAC1 or HDAC3, are increased in hippocampal and entorhinal cortex neurons in AD compared to HDAC2 in control subjects (Graff et al., 2012). Western blots of frontal cortex revealed significant increases in HDAC1, HDAC3, HDAC4, and HDAC6 in MCI and mAD compared to NCI whereas HDAC2 levels remained stable (Mahady et al., 2018). These findings suggest that differential epigenetic regulation occurs across brain regions affected in AD.

## Cholinotrophic Biomarkers for the Progression of AD

As discussed above, protein levels of proNGF are increased in postmortem neocortex (Peng et al., 2004,) and hippocampus (Mufson et al., 2012b) of subjects who died with a clinical diagnosis of MCI or mild AD compared to NCI, respectively, which correlated with poorer antemortem cognitive test scores (Peng et al., 2004; Mufson et al., 2012b). These observations initiated an investigation of whether altered CSF levels of proNGF mark a transition from NCI to MCI and AD. Ventricular CSF (vCSF) was obtained postmortem from RROS participants and premortem lumbar CSF was collected from subjects clinically diagnosed as CDR 0 (no dementia), CDR 0.5 (MCI or very mild AD), or CDR 1 (mild AD) at the Washington University Knight AD Research Center (Counts et al., 2016). Quantitative western blotting of vCSF revealed a significant (50%) increase in proNGF levels in aMCI compared to NCI and a 70% increase in AD compared to NCI, which displayed a significant inverse relationship between increasing vCSF proNGF levels and cognitive deterioration (Counts et al., 2016). Lumbar CSF proNGF levels were significantly (30%) increased in CDR 0.5 and CDR 1 compared to CDR 0 cases. Although no difference in levels of Aβ_1–42_, total tau, phospho-tau, or phospho-tau/Aβ_1–42_ was found between groups, the ratio of total tau/Aβ_1–42_ levels was 50% higher in CDR 1 compared to CDR 0 subjects. Ratios were calculated for proNGF/Aβ_1–42_, proNGF/total tau, and proNGF/phospho-tau to determine if the inclusion of CSF proNGF levels improved the reliability of these biomarkers. Interestingly, proNGF/Aβ_1–42_ levels were 50% higher in CDR 0.5 and CDR 1 compared to CDR 0, whereas proNGF/total tau and proNGF/phospho-tau were unchanged between groups, suggesting the inclusion of proNGF as a candidate biomarker will improve the diagnostic probability needed to identify people in the preclinical or prodromal stages of AD.

## NGF Therapy as a Treatment Strategy for AD

**FIGURE 11:**
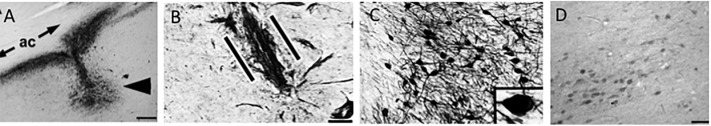
**(A)** Nerve growth factor (NGF) immunolabeling shows the site of NGF gene delivery in the human nucleus basalis of Meynert (arrowhead) under the anterior commissure (ac). Scale bar = 325 μm. The patient received the injection 3 years previously. **(B)** p75^NTR^ neurotrophin receptor immunolabeling shows basal forebrain cholinergic axons penetrating into the graft (between the parallel lines) in a linear fashion. Scale bar = 25 μm. **(C)** Nerve growth-factor-expressing neurons compared with less intense labeling in the nucleus basalis of Meynert neurons located 3 mm from the injection site **(D)**. Scale bar in **(C)** = 100 μm and in **(D)** = 325 μm. Images reproduced from Tuszynski et al. (2015).

Evidence derived from studies employing NGF as a treatment strategy to rescue the cholinotrophic cortical projection system has revealed some promising results regarding prevention of CBF neuron atrophy and a correction of behavioral deficits resulting from experimental damage or normal aging (Hefti, 1986; Williams et al., 1986; Hartikka and Hefti, 1988; Hatanaka et al., 1988; Nabeshima et al., 1994; Burgos et al., 1995; Charles et al., 1996; Humpel and Weis, 2002). This evidence led to the concept that treatments that facilitate NGF would be beneficial in reversing cholinotrophic dysfunction in AD. However, examination of studies of early NGF systemic administration showed several weaknesses including bioavailability of the neurotrophin to reach target neurons, unregulated neurotransmitter release, hyperinnervation, sprouting of neurons, sympathetic stimulation, induction of antibodies, cachexia, and hyperalgesia (Sramek and Cutler, 1999; Jonhagen, 2000; McArthur et al., 2000; Apfel, 2001). However, after further testing in rat and non-human primate animal models (Gnahn et al., 1983; Hefti, 1986; Gage et al., 1989, 1990; Hefti et al., 1989; Higgins et al., 1989; Tuszynski et al., 1990, 1991, 1996; Blesch and Tuszynski, 1995) and taking into account past failures (e.g., poor drug delivery and unwanted systemic side effects), a Phase I clinical trial was undertaken to determine the utility of *ex vivo* NGF gene therapy for AD (Tuszynski et al., 2005). The goal was both to protect cholinotrophic neurons within the nbM from degeneration as well as augment the function of remaining cholinergic neurons by intracranial delivery of human NGF. During clinical trials, patients with AD, underwent NGF transcript therapy using *ex vivo* or *in vivo* gene transfer directed at the cholinergic neurons within the nbM. Degenerating nbM neurons were found to respond to NGF, with axonal sprouting toward the NGF source (Figure 11). Participants that had unilateral gene transfer displayed neuronal hypertrophy in the NGF-treated cholinergic nbM.

Moreover, patients that sustained adeno-associated viral vector (serotype 2)-mediated NGF gene transfer, displayed activation of cellular signaling and functional markers. Interestingly, nbM neurons that exhibited pathologic tau and those that were tau immunonegative both expressed NGF, indicating that tangle-bearing neurons can be infected with therapeutic genes, which activate cell signaling. No adverse effects related to NGF treatment were found in these studies. In summary, these findings revealed that degenerating neurons can respond to NGF with axonal sprouting, cell hypertrophy, and activation of functional markers. These studies demonstrated that NGF-induced sprouting persisted for 10 years post NGF gene transfer and that this therapy appears safe over long periods of time (Tuszynski et al., 2015). It should be kept in mind that these studies were not double-blind, placebo-controlled clinical trials. Further clinical investigation of gene therapy approaches is warranted.

## Small Molecule Neurotrophin Compounds for Treatment of AD

Small molecule partial agonist and antagonist activators of NGF receptors have been considered for the treatment of AD (Skaper, 2008). For example, a high-throughput screening assay of small-molecule agonists for TrkA identified gambogic amide, an alkaloid used in traditional Chinese medicine as a possible candidate (Jang et al., 2007). Gambogic amide binds selectively to TrkA (but not TrkB and TrkC), phosphorylates TrkA tyrosine residues, and activates the Akt and Erk TrkA-mediated NGF signaling pathways. Gambogic amide has been demonstrated to ameliorate excitotoxic damage and promote neurite outgrowth in PC12 cells and reduce kainic acid neuronal induced cell death in mice (Jang et al., 2007).

**FIGURE 12:**
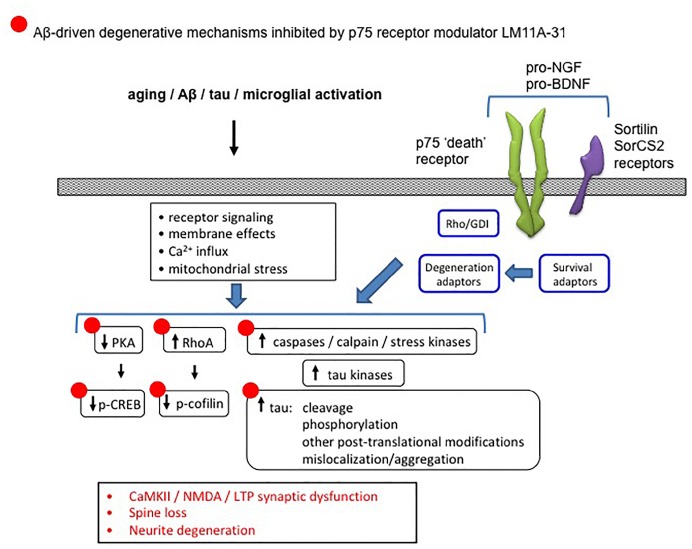
Diagram showing the association between p75^NTR^ and Alzheimer’s-related degenerative signaling networks. Aging, Aβ and other processes produce numerous changes in signaling pathways involved in neurite and synapse function and degeneration. Degenerative signaling promoted by p75^NTR^ in either its unliganded state, or in response to proneurotrophin binding to p75^NTR^ and its sortilin family co-receptors, and mediated through one or more receptor adaptors enables or promotes AD-related degenerative processes. LM11A-31, which interferes with proneurotrophin degenerative signaling and promotes supportive/survival signaling through p75^NTR^, reverses many of these AD-associated effects (indicated by •).

Several lines of evidence also point to the modulation of degenerative signaling promoted by p75^NTR^ as a potential therapeutic target for AD. Through either its constitutive activity in the unliganded state, or via that stimulated by its proneurotropin (proNT) ligands, p75^NTR^ promotes degenerative signaling mechanisms including activation of JNK, caspase, and RhoA (Casaccia-Bonnefil et al., 1996; Harrington et al., 2002; Troy et al., 2002; Ibanez and Simi, 2012; Coulson and Nykjaer, 2013) each of which likely contributes to AD-related degeneration (Figure 12). Crossing various AD mouse models with a p75^NTR^ knockout mouse construct results in reduced neuronal degeneration (Sotthibundhu et al., 2008; Knowles et al., 2009; Murphy et al., 2015). Multiple studies have identified genetic polymorphisms in the genes encoding p75^NTR^, proNGF, proBDNF, or p75^NTR^ co-receptors including sortilin and SorCS2 that mediate proNT binding associated with increased AD risk (Cozza et al., 2008; Di Maria et al., 2012; Anastasia et al., 2013; Reitz et al., 2013; Andersson et al., 2016; Matyi et al., 2017). The first small molecule ligands found to interact with p75^NTR^ and modulate its signaling were identified using an *in silico* screening strategy based on synthetic peptides modeled on the loop I domain of NGF and mutational analyses of neurotrophin ligands (Massa et al., 2006). Two prototype small molecule compounds, LM11A-31 and LM11A-24, were found to prevent neuronal death in cell culture conditions under which neuronal survival is dependent on the addition of neurotrophins. These small molecule ligands effectively acted as positive p75^NTR^ modulators, inhibited proNGF-induced cell death (Massa et al., 2006) and blocked binding of proNGF to p75^NTR^ (Tep et al., 2013). Under these conditions, small molecule ligands could be described as p75^NTR^ ‘antagonists.’ LM11A-31 has been shown to stimulate recruitment of interleukin-1 receptor-associated kinase (IRAK) survival adaptor to p75^NTR^ and to upregulate downstream NF-κB and Akt pro-survival signaling. Neurotrophic activity and signaling were absent in cultures using p75^NTR^−/− neurons or in the presence of p75^NTR^-ECD blocking antibody (Massa et al., 2006). LM11A-31 blocks proNGF-induced degenerative mechanisms in models of spinal cord injury (Tep et al., 2013), neurogenic bladder dysfunction (Ryu et al., 2018), and arthritis (Minnone et al., 2017).

Multiple preclinical studies indicate a role for small molecule modulation of p75^NTR^ as a therapeutic approach for slowing progression of AD-related degenerative mechanisms. Intracellular signaling networks linked to p75^NTR^ have substantial integration with degenerative signaling networks implicated in AD (Nguyen et al., 2014). In a model of AD in which cultured hippocampal neurons are exposed to Aβ oligomers, LM11A-31 and LM11A-24 inhibited the following Aβ-induced degenerative mechanisms: activation of calpain/cdk5, GSK3β, JNK c-Jun, p38 kinase, RhoA; excessive tau phosphorylation; and inactivation of Akt and CREB (Yang et al., 2008). These ligands also blocked Aβ-induced neuritic dystrophy and hippocampal long-term potentiation (LTP) impairment. In studies employing the hAPP^Lond/Swe^ AD mouse model, a once-daily administration of LM11A-31 over 3 months corrected behavioral deficits and inhibited neurodegenerative molecular and cellular pathology including tau phosphorylation and misfolding, neurite dystrophy, microglial activation and astrocyte activation (Knowles et al., 2013; Nguyen et al., 2014). However, there was no effect on lowering soluble Aβ or amyloid plaque levels, consistent with a mechanism in which modulation of p75^NTR^ inhibits the ability of Aβ to promote neural degeneration including tau-related molecular degenerative events and synaptic failure. The ability of LM11A-31 to reduce measures of microglial activation in hAPP^Lond/Swe^ AD mice was confirmed using multiple markers of microglial activation as well as micro-PET imaging using a PET ligand directed to the translocator protein (TSPO) (James et al., 2017). In hAPP^Lond/Swe^ AD mouse studies employing treatment in late-stage, application of LM11A-31 resulted in a partial reversal of neural degeneration, perhaps indicative of a particularly robust biological effect (Simmons et al., 2014). Figure 12 summarizes the ability of the p75^NTR^ modulator LM11A-31, which interferes with proNT degenerative signaling and promotes supportive/survival signaling through p75^NTR^, to inhibit/reverse AD degenerative mechanisms.

Based on *in vivo* preclinical studies, a modified formulation of LM11A-31, as a first-in-class compound directed against a novel target, was tested in a phase 1 trial in normal subjects and found to be safe. This compound is currently in a randomized, double-blinded, phase 2a exploratory endpoint trial in subjects with mild to moderate AD (NCT03069014). Treatment is administered via daily oral capsules for 6 months, and measurements at baseline and post-treatment include the following: cognitive testing with multiple batteries; MRI volumetric measures and FDG-PET imaging; and CSF AD core biomarkers along with biomarkers relevant to target engagement and mechanisms of action.

## Concluding Comments

**FIGURE 13:**
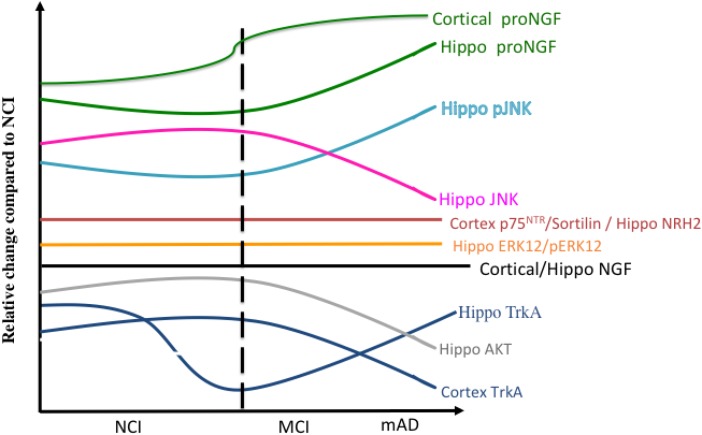
Summary diagram showing the changes in the relative levels of NGF-related proteins in the cortex and hippocampus during the progression of AD.

Figure 13 summarizes the relative changes of NGF upstream and downstream signaling pathways within the cortex and hippocampus during the progression of AD. A preponderance of data indicates that normative levels of NGF and its cognate receptors are required for the survival and maintenance of the cholinotrophic system. The preservation of cholinergic nbM neurons in the face of reduced numbers of TrkA and p75^NTR^-positive neurons in MCI and mild AD indicates that there is not a frank loss of cholinergic perikarya *per se* but a phenotypic downregulation of receptor proteins early in the disease process. Both transcript and protein data indicate that CBF neuron dysfunction is associated with an imbalance between TrkA-mediated survival signaling and proNGF/p75^NTR^-mediated pro-apoptotic signaling. Despite these degenerative events, the cholinotrophic system is capable of cellular resilience and/or neuroplasticity during the prodromal (DeKosky et al., 2002) and even the later stages of the disease (Mufson et al., 2012b). In addition to neurotrophic dysfunction, alterations in nuclear epigenetic proteins occur within cholinotrophic nbM neurons during the progression of AD, particularly HDAC2, suggesting a mechanism associated with changes in transcript expression. Increased proNGF quantified in postmortem vCSF or premortem lumbar CSF marked the transition to MCI and to AD, suggesting that this proneurotrophin is a useful biomarker of disease progression. Clinical trials provide evidence that NGF gene therapy has the potential to be a treatment approach for the prevention of CBF degeneration in AD. Perhaps combining this therapeutic approach with the development of small molecule agonists to TrkA to facilitate prosurvival signaling (Jang et al., 2007) or small molecule antagonists to p75^NTR^ for anti-apoptotic actions should be added to the treatment toolbox for MCI and AD.

## Author Contributions

EJM designed and wrote the manuscript. SEC, SDG, LM, SEP, SMM, FML, and MDI contributed text and editing of the manuscript.

## Conflict of Interest Statement

EJM consults for RegenxBio. MDI discloses consultant fees at GE Healthcare. FML and SMM are listed as inventors on patents relating to LM11A-31, which are assigned to the University of North Carolina, University of California (UC), San Francisco and the Department of Veterans Affairs (VA). FML and SMM are entitled to royalties distributed by UC and the VA per their standard agreements. FML is a principal of, and has a financial interest in PharmatrophiX, a company focused on the development of small molecule ligands for neurotrophin receptors which has licensed several of these patents. The funders had no role in study design, data collection and analysis, decision to publish, or preparation of the manuscript. The remaining authors declare that the research was conducted in the absence of any commercial or financial relationships that could be construed as a potential conflict of interest.

## References

[B1] Aizawa S. Teramoto K. Yamamuro Y. (2012). Histone deacetylase 9 as a negative regulator for choline acetyltransferase gene in NG108-15 neuronal cells. *Neuroscience* 205 63–72. 10.1016/j.neuroscience.2011.12.024 22226696

[B2] Aizawa S. Yamamuro Y. (2010). Involvement of histone acetylation in the regulation of choline acetyltransferase gene in NG108-15 neuronal cells. *Neurochem. Int.* 56 627–633. 10.1016/j.neuint.2010.01.007 20100532

[B3] Allen S. J. MacGowan S. H. Treanor J. J. S. Feeney R. Wilcock G. K. Dawbarn D. (1991). Normal B-NGF content in Alzheimer’s disease cerebral cortex and hippocampus. *Neurosci. Lett.* 131 135–139. 10.1016/0304-3940(91)90354-v 1791973

[B4] Al-Shawi R. Hafner A. Olsen J. Chun S. Raza S. Thrasivoulou C. (2008). Neurotoxic and neurotrophic roles of proNGF and the receptor sortilin in the adult and ageing nervous system. *Eur. J. Neurosci.* 27 2103–2114. 10.1111/j.1460-9568.2008.06152.x 18412630

[B5] Alzheimer A. (1911). Uber eigenartige krankheitsfaelle des spaetern alters, zeitschrift fuer die gesamte. *Neurol. Psych.* 4 256–286.

[B6] Alzheimer’s Association (2019). 2018 Alzheimer’s disease facts and figures. *Alzheimer’s Dement.* 14 367–429. 10.1016/j.jalz.2018.02.001 21414557

[B7] Amadoro G. Corsetti V. Ciotti M. T. Florenzano F. Capsoni S. Amato G. (2011). Endogenous abeta causes cell death via early tau hyperphosphorylation. *Neurobiol. Aging* 32 969–990. 10.1016/j.neurobiolaging.2009.06.005 19628305

[B8] Anastasia A. Deinhardt K. Chao M. V. Will N. E. Irmady K. Lee F. S. (2013). Val66Met polymorphism of BDNF alters prodomain structure to induce neuronal growth cone retraction. *Nat. Commun.* 4:2490. 10.1038/ncomms3490 24048383 PMC3820160

[B9] Andersson C. Hansson O. Minthon L. Andreasen N. Blennow K. Zetterberg H. (2016). A genetic variant of the sortilin 1 gene is associated with reduced risk of alzheimer’s disease. *J. Alzheimers Dis.* 53 1353–1363. 10.3233/JAD-160319 27392867 PMC5147507

[B10] Apfel S. C. (2001). Neurotrophic factor therapy–prospects and problems. *Clin. Chem. Lab. Med.* 39 351–355. 11388661 10.1515/CCLM.2001.055

[B11] Arriagada P. Growdon J. Hedley-Whyte E. Hyman B. (1992). Neurofibrillary tangles but not senile plaques parallel duration and severity of Alzheimer’s disease. *Neurology* 42 631–639. 1549228 10.1212/wnl.42.3.631

[B12] Babu J. R. Geetha T. Wooten M. W. (2005). Sequestosome 1/p62 shuttles polyubiquitinated tau for proteasomal degradation. *J. Neurochem.* 94 192–203. 10.1111/j.1471-4159.2005.03181.x 15953362

[B13] Ballinger E. C. Ananth M. Talmage D. A. Role L. W. (2016). Basal forebrain cholinergic circuits and signaling in cognition and cognitive decline. *Neuron* 91 1199–1218. 10.1016/j.neuron.2016.09.006 27657448 PMC5036520

[B14] Bamji S. X. Majdan M. Pozniak C. D. Belliveau D. J. Aloyz R. Kohn J. (1998). The p75 neurotrophin receptor mediates neuronal apoptosis and is essential for naturally occurring sympathetic neuron death. *J. Cell Biol.* 140 911–923. 10.1083/jcb.140.4.911 9472042 PMC2141754

[B15] Bartus R. T. Dean R. L. III Beer B. Lippa A. S. (1982). The cholinergic hypothesis of geriatric memory dysfunction. *Science* 217 408–414. 10.1126/science.7046051 7046051

[B16] Bekdash R. (2016). “Choline and the Brain: An Epigenetic Perspective,” in *The Benefits of Natural Products for Neurodegenerative Diseases*, eds EssaM A. M. Guillemin G. (Cham: Springer), 381–399. 10.1007/978-3-319-28383-8_21 27651265

[B17] Berger Z. Roder H. Hanna A. Carlson A. Rangachari V. Yue M. (2007). Accumulation of pathological tau species and memory loss in a conditional model of tauopathy. *J. Neurosci.* 27 3650–3662. 10.1523/JNEUROSCI.0587-07.2007 17409229 PMC6672413

[B18] Bertrand J. Plouffe V. Senechal P. Leclerc N. (2010). The pattern of human tau phosphorylation is the result of priming and feedback events in primary hippocampal neurons. *Neuroscience* 168 323–334. 10.1016/j.neuroscience.2010.04.009 20394726

[B19] Blesch A. Tuszynski M. (1995). Ex vivo gene therapy for Alzheimer’s disease and spinal cord injury. *Clin. Neurosci.* 3 268–274. 8914793

[B20] Blocq P. Marinesco G. (1892). Sur les lésions et la pathogénie de l’épilepsie dite essentielle. *Sem. Med.* 12 445–456.

[B21] Braak H. Braak E. (1991). Neuropathological stageing of Alzheimer-related changes. *Acta Neuropathol.* 82 239–259. 10.1007/bf00308809 1759558

[B22] Bronfman F. C. Fainzilber M. (2004). Multi-tasking by the p75 neurotrophin receptor: sortilin things out? *EMBO Rep.* 5 867–871. 10.1038/sj.embor.7400219 15470383 PMC1299130

[B23] Bruno M. A. Clarke P. B. Seltzer A. Quirion R. Burgess K. Cuello A. C. (2004). Long-lasting rescue of age-associated deficits in cognition and the CNS cholinergic phenotype by a partial agonist peptidomimetic ligand of TrkA. *J. Neurosci.* 24 8009–8018. 10.1523/jneurosci.1508-04.2004 15371501 PMC6729798

[B24] Bruno M. A. Counts S. E. Mufson E. J. Cuello A. C. (2007). Increased MMP-9 cortical level and activity in subjects with Mild cognitive Impairment. *Soc. Neurosci.* 68 1309–1318.

[B25] Bruno M. A. Cuello A. C. (2006). Activity-dependent release of precursor nerve growth factor, conversion to mature nerve growth factor, and its degradation by a protease cascade. *Proc. Natl. Acad. Sci. U.S.A.* 103 6735–6740. 10.1073/pnas.0510645103 16618925 PMC1458950

[B26] Bruno M. A. Leon W. C. Fragoso G. Mushynski W. E. Almazan G. Cuello A. C. (2009). Amyloid beta-induced nerve growth factor dysmetabolism in Alzheimer disease. *J. Neuropathol. Exp. Neurol.* 68 857–869. 10.1097/NEN.0b013e3181aed9e6 19606067

[B27] Burgos I. Cuello A. C. Liberini P. Pioro E. Masliah E. (1995). NGF-mediated synaptic sprouting in the cerebral cortex of lesioned primate brain. *Brain Res.* 692 154–160. 10.1016/0006-8993(95)00696-n 8548299

[B28] Canu N. Amadoro G. Triaca V. Latina V. Sposato V. Corsetti V. (2017). The intersection of NGF/TrkA signaling and amyloid precursor protein processing in alzheimer’s disease neuropathology. *Int. J. Mol. Sci.* 18:1319. 10.3390/ijms18061319 28632177 PMC5486140

[B29] Capsoni S. Ugolini G. Comparini A. Ruberti F. Berardi N. Cattaneo A. (2000). Alzheimer-like neurodegeneration in aged antinerve growth factor transgenic mice. *Proc. Natl. Acad. Sci. U.S.A.* 97 6826–6831. 10.1073/pnas.97.12.6826 10841577 PMC18754

[B30] Casaccia-Bonnefil P. Carter B. D. Dobrowsky R. T. Chao M. V. (1996). Death of oligodendrocytes mediated by the interaction of nerve growth factor with its receptor p75. *Nature* 383 716–719. 10.1038/383716a0 8878481

[B31] Cavedo E. Grothe M. Colliot O. Lista S. Chupin M. Dormont D. (2017). Reduced basal forebrain atrophy progression in a randomized donepezil trial in prodromal Alzheimer’s disease. *Sci. Rep.* 7 1–10. 28916821 10.1038/s41598-017-09780-3 PMC5601919

[B32] Chao M. (2003). Neurotrophins and their receptors: a convergence point for many signalling pathways. *Nat. Rev. Neurosci.* 4 299–309. 10.1038/nrn1078 12671646

[B33] Charles V. Mufson E. J. Friden P. M. Bartus R. T. Kordower J. H. (1996). Atrophy of cholinergic basal forebrain neurons following excitotoxic cortical lesions is reversed by intravenous administration of an NGF conjugate. *Brain Res.* 728 193–203. 10.1016/s0006-8993(96)00398-8 8864482

[B34] Chen S. Owens G. C. Makarenkova H. Edelman D. B. (2010). HDAC6 regulates mitochondrial transport in hippocampal neurons. *PLoS One* 5:e10848. 10.1371/journal.pone.0010848 20520769 PMC2877100

[B35] Christensen D. D. (2007). Alzheimer’s disease: progress in the development of anti-amyloid disease-modifying therapies. *CNS Spectr.* 12 113–116, 119–123. 17277711 10.1017/s1092852900020629

[B36] Cook C. Gendron T. F. Scheffel K. Carlomagno Y. Dunmore J. DeTure M. (2012). Loss of HDAC6, a novel CHIP substrate, alleviates abnormal tau accumulation. *Hum. Mol. Genet.* 21 2936–2945. 10.1093/hmg/dds125 22492994 PMC3373241

[B37] Coulson E. Nykjaer A. (2013). Up-regulation of sortilin mediated by amyloid-β and p75NTR: safety lies in the middle course. *J. Neurochem.* 127 149–151. 10.1111/jnc.12389 23991915

[B38] Counts S. E. Che S. Ginsberg S. D. Mufson E. J. (2011). Gender differences in neurotrophin and glutamate receptor expression in cholinergic nucleus basalis neurons during the progression of Alzheimer’s disease. *J. Chem. Neuroanat.* 42 111–117. 10.1016/j.jchemneu.2011.02.004 21397006 PMC3155625

[B39] Counts S. E. He B. Prout J. G. Michalski B. Farotti L. Fahnestock M. (2016). Cerebrospinal fluid proNGF: a putative biomarker for early Alzheimer’s disease. *Curr. Alzheimer Res.* 13 800–808. 10.2174/1567205013666160129095649 26825093 PMC5827942

[B40] Counts S. E. Nadeem M. Wuu J. Ginsberg S. D. Saragovi H. U. Mufson E. J. (2004). Reduction of cortical TrkA but not p75(NTR) protein in early-stage Alzheimer’s disease. *Ann. Neurol.* 56 520–531. 10.1002/ana.20233 15455399

[B41] Cozza A. Melissari E. Iacopetti P. Mariotti V. Tedde A. Nacmias B. (2008). SNPs in neurotrophin system genes and Alzheimer’s disease in an Italian population. *J. Alzheimers Dis.* 15 61–70. 10.3233/jad-2008-15105 18780967

[B42] Crutcher K. A. Scott S. A. Liang S. Everson W. V. Weingartner J. (1993). Detection of NGF-like activity in human brain tissue: Increased levels in Alzheimer’s disease. *J. Neurosci.* 13 2540–2550. 10.1523/jneurosci.13-06-02540.1993 8501520 PMC6576515

[B43] Dawson M. A. Kouzarides T. (2012). Cancer epigenetics: from mechanism to therapy. *Cell* 150 12–27. 10.1016/j.cell.2012.06.013 22770212

[B44] DeKosky S. T. Ikonomovic M. D. Styren S. D. Beckett L. Wisniewski S. Bennett D. A. (2002). Upregulation of choline acetyltransferase activity in hippocampus and frontal cortex of elderly subjects with mild cognitive impairment. *Ann. Neurol.* 51 145–155. 10.1002/ana.10069 11835370

[B45] Di Maria E. Giorgio E. Uliana V. Bonvicini C. Faravelli F. Cammarata S. (2012). Possible influence of a non-synonymous polymorphism located in the NGF precursor on susceptibility to late-onset Alzheimer’s disease and mild cognitive impairment. *J. Alzheimers Dis.* 29 699–705. 10.3233/JAD-2012-112006 22330829

[B46] Ding H. Dolan P. J. Johnson G. V. (2008). Histone deacetylase 6 interacts with the microtubule-associated protein tau. *J. Neurochem.* 106 2119–2130. 10.1111/j.1471-4159.2008.05564.x 18636984 PMC2574575

[B47] Douchamps V. Mathis C. (2017). A second wind for the cholinergic system in Alzheimer’s therapy. *Behav. Pharmacol.* 28 112–123. 10.1097/FBP.0000000000000300 28240674

[B48] Edwards R. H. Selby M. J. Garcia P. D. Rutter W. J. (1988). Processing of the native nerve growth factor precursor to form biologically active nerve growth factor. *J. Biol. Chem.* 263 6810–6815. 3360808

[B49] Fahnestock M. Michalski B. Xu B. Coughlin M. (2001). The precursor pro-nerve growth factor is the predominant form of nerve growth factor in brain and is increased in Alzheimer’s disease. *Mol. Cell Neurosci.* 18 210–220. 10.1006/mcne.2001.1016 11520181

[B50] Fahnestock M. Scott S. A. Jette N. Weingartner J. A. Crutcher K. A. (1996). Nerve growth factor mRNA and protein levels measured in the same tissue from normal and Alzheimer’s disease parietal cortex. *Brain Res. Mol. Brain Res.* 42 175–178. 10.1016/s0169-328x(96)00193-3 8915599

[B51] Fahnestock M. Yu G. Michalski B. Mathew S. Colquhoun A. Ross G. M. (2004). The nerve growth factor precursor proNGF exhibits neurotrophic activity but is less active than mature nerve growth factor. *J. Neurochem.* 89 581–592. 10.1111/j.1471-4159.2004.02360.x 15086515

[B52] Frade J. M. (2000). Unscheduled re-entry into the cell cycle induced by NGF precedes cell death in nascent retinal neurones. *J. Cell Sci.* 113(Pt 7), 1139–1148. 10704365 10.1242/jcs.113.7.1139

[B53] Francke U. Martinville B. D. Coussens L. Ullrich A. (1983). The human gene for the beta subunit of nerve growth factor is located on the proximal short arm of chromosome 1. *Science* 222 1248–1251. 10.1126/science.6648531 6648531

[B54] Friedman W. J. (2000). Neurotrophins induce death of hippocampal neurons via the p75 receptor. *J. Neurosci.* 20 6340–6346. 10.1523/jneurosci.20-17-06340.2000 10964939 PMC6772976

[B55] Gage F. H. Rosenberg M. B. Tuszynski M. H. Yoshida K. Armstrong D. M. Hayes R. C. (1990). Gene therapy in the CNS: intracerebral grafting of genetically modified cells. *Prog. Brain Res.* 86 205–217. 10.1016/s0079-6123(08)63178-7 2087558

[B56] Gage F. H. Tuszynski M. H. Chen K. S. Armstrong D. Buzsaki G. (1989). Survival, growth and function of damaged cholinergic neurons. *EXS* 57 259–274. 10.1007/978-3-0348-9138-7_26 2533097

[B57] Gilmor M. L. Erickson J. D. Varoqui H. Hersh L. B. Bennett D. A. Cochran E. J. (1999). Preservation of nucleus basalis neurons containing choline acetyltransferase and the vesicular acetylcholine transporter in the elderly with mild cognitive impairment and early Alzheimer’s disease. *J. Comp. Neurol.* 411 693–704. 10.1002/(sici)1096-9861(19990906)411 10421878

[B58] Ginsberg S. Malek-Ahmadi M. Alldred M. Che S. Elarova I. Chen Y. (2019). Selective decline of neurotrophin and neurotrophin receptor genes within CA1 pyramidal neurons and hippocampus proper: correlation with cognitive performance and neuropathology in mild cognitive impairment and Alzheimer’s disease. *Hippocampus* 29 422–439. 10.1002/hipo.22802 28888073 PMC5844851

[B59] Ginsberg S. Martin L. (1998). Ultrastructural analysis of the progression of neurodegeneration in the septum following fimbria–fornix transection. *Neuroscience* 86 1259–1272. 10.1016/s0306-4522(98)00136-5 9697131

[B60] Ginsberg S. D. Alldred M. J. Counts S. E. Cataldo A. M. Neve R. L. Jiang Y. (2010). Microarray analysis of hippocampal CA1 neurons implicates early endosomal dysfunction during Alzheimer’s disease progression. *Biol. Psychiatry* 68 885–893. 10.1016/j.biopsych.2010.05.030 20655510 PMC2965820

[B61] Ginsberg S. D. Che S. Counts S. E. Mufson E. J. (2006a). Shift in the ratio of three-repeat tau and four-repeat tau mRNAs in individual cholinergic basal forebrain neurons in mild cognitive impairment and Alzheimer’s disease. *J. Neurochem.* 96 1401–1408. 10.1111/j.1471-4159.2005.03641.x 16478530

[B62] Ginsberg S. D. Che S. Wuu J. Counts S. E. Mufson E. J. (2006b). Down regulation of trk but not p75NTR gene expression in single cholinergic basal forebrain neurons mark the progression of Alzheimer’s disease. *J. Neurochem.* 97 475–487. 10.1111/j.1471-4159.2006.03764.x 16539663

[B63] Ginsberg S. D. Hemby S. E. Lee V. M. Eberwine J. H. Trojanowski J. Q. (2000). Expression profile of transcripts in Alzheimer’s disease tangle-bearing CA1 neurons. *Ann. Neurol.* 48 77–87. 10.1002/1531-8249(200007)48 10894219

[B64] Gnahn H. Hefti F. Heumann R. Schwab M. E. Thoenen H. (1983). NGF-mediated increase of choline acetyltransferase (ChAT) in the neonatal rat forebrain: evidence for a physiological role of NGF in the brain? *Brain Res.* 285 45–52. 10.1016/0165-3806(83)90107-4 6136314

[B65] Goedert M. Fine A. Dawbarn D. Wilcock G. K. Chao M. V. (1989). Nerve growth factor receptor mRNA distribution in human brain: normal levels in basal forebrain in Alzheimer’s disease. *Mol. Brain Res.* 5 1–7. 10.1016/0169-328x(89)90011-9 2538704

[B66] Goedert M. Hasegawa M. Jakes R. Lawler S. Cuenda A. Cohen P. (1997). Phosphorylation of microtubule-associated protein tau by stress-activated protein kinases. *FEBS Lett.* 409 57–62. 10.1016/s0014-5793(97)00483-3 9199504

[B67] Graff J. Rei D. Guan J. S. Wang W. Y. Seo J. Hennig K. M. (2012). An epigenetic blockade of cognitive functions in the neurodegenerating brain. *Nature* 483 222–226. 10.1038/nature10849 22388814 PMC3498952

[B68] Graff J. Tsai L. H. (2013). Histone acetylation: molecular mnemonics on the chromatin. *Nat. Rev. Neurosci.* 14 97–111. 10.1038/nrn3427 23324667

[B69] Grothe M. Heinsen H. Teipel S. J. (2012). Atrophy of the cholinergic Basal forebrain over the adult age range and in early stages of Alzheimer’s disease. *Biol. Psychiatry* 71 805–813. 10.1016/j.biopsych.2011.06.019 21816388 PMC3701122

[B70] Guan J. S. Haggarty S. J. Giacometti E. Dannenberg J. H. Joseph N. Gao J. (2009). HDAC2 negatively regulates memory formation and synaptic plasticity. *Nature* 459 55–60. 10.1038/nature07925 19424149 PMC3498958

[B71] Guillozet A. L. Weintraub S. Mash D. C. Mesulam M. M. (2003). Neurofibrillary tangles, amyloid, and memory in aging and mild cognitive impairment. *Arch. Neurol.* 60 729–736. 10.1001/archneur.60.5.729 12756137

[B72] Hampel H. Mesulam M. M. Cuello A. C. Farlow M. R. Giacobini E. Grossberg G. T. (2018). The cholinergic system in the pathophysiology and treatment of Alzheimer’s disease. *Brain* 141 1917–1933. 10.1093/brain/awy132 29850777 PMC6022632

[B73] Hampel H. Schneider L. S. Giacobini E. Kivipelto M. Sindi S. Dubois B. (2015). Advances in the therapy of Alzheimer’s disease: targeting amyloid beta and tau and perspectives for the future. *Expert. Rev. Neurother.* 15 83–105. 10.1586/14737175.2015.995637 25537424

[B74] Hardy J. Selkoe D. J. (2002). The amyloid hypothesis of Alzheimer’s disease: progress and problems on the road to therapeutics. *Science* 297 353–356. 10.1126/science.1072994 12130773

[B75] Harrington A. Kim J. Y. Yoon S. (2002). Activation of Rac GTPase by p75 is necessary for c-jun N-terminal kinase-mediated apoptosis. *J. Neurosci.* 22 156–166. 10.1523/jneurosci.22-01-00156.2002 11756498 PMC6757583

[B76] Hartikka J. Hefti F. (1988). Comparison of nerve growth factor’s effects on development of septum, striatum, and nucleus basalis cholinergic neurons in vitro. *J. Neurosci. Res.* 21 352–364. 10.1002/jnr.490210227 3216428

[B77] Hatanaka H. Nihonmatsu I. Tsukui H. (1988). Nerve growth factor promotes survival of cultured magnocellular cholinergic neurons from nucleus basalis of Meynert in postnatal rats. *Neurosci. Lett.* 90 63–68. 10.1016/0304-3940(88)90787-2 2842704

[B78] Hefti F. (1986). Nerve growth factor promotes survival of septal cholinergic neurons after fimbrial transections. *J. Neurosci.* 6 2155–2162. 10.1523/jneurosci.06-08-02155.1986 3746405 PMC6568758

[B79] Hefti F. Hartikka J. Knusel B. (1989). Function of neurotrophic factors in the adult and aging brain and their possible use in the treatment of neurodegenerative diseases. *Neurobiol. Aging* 10 515–533. 10.1016/0197-4580(89)90118-8 2682327

[B80] Hefti F. Mash D. C. (1989). Localization of nerve growth factor receptors in the normal human brain and in Alzheimer’s disease. *Neurobiol. Aging* 10 75–87. 10.1016/s0197-4580(89)80014-4 2547172

[B81] Hellweg R. Gericke C. A. Jendroska K. Hartung H. D. Cervos-Navarro J. (1998). NGF content in the cerebral cortex of non-demented patients with amyloid-plaques and in symptomatic Alzheimer’s disease. *Int. J. Dev. Neurosci.* 16 787–794. 10.1016/s0736-5748(98)00088-4 10198825

[B82] Henry J. M. (1998). Neurons and nobel prizes: a centennial history of neuropathology. *Neurosurgery* 42 143–155; discussion155–146. 9442516 10.1097/00006123-199801000-00031

[B83] Higgins G. A. Koh S. Chen K. S. Gage F. H. (1989). NGF induction of NGF receptor gene expression and cholinergic neuronal hypertrophy within the basal forebrain of the adult rat. *Neuron* 3 247–256. 10.1016/0896-6273(89)90038-x 2560393

[B84] Ho A. Moore R. Lopez O. Kuller L. Becker J. (2008). Basal forebrain atrophy is a presymptomatic marker for alzheimer’s disease. *Alzheimers Dement.* 4 271–279. 10.1016/j.jalz.2008.04.005 18631978 PMC2517158

[B85] Hock C. Heese K. Muller-Spahn F. Huber P. Riesen W. Nitsch R. M. (2000). Increased CSF levels of nerve growth factor in patients with Alzheimer’s disease. *Neurology* 54 2009–2011. 10.1212/wnl.54.10.2009 10822447

[B86] Humpel C. Weis C. (2002). “Nerve growth factor and cholinergic CNS neurons studied in organotypic brain slices,” in *Ageing and Dementia Current and Future Concepts. Journal of Neural Transmission* Vol. 62 eds Jellinger K. A. Schmidt R. Windisch M. (Vienna: Springer). 10.1007/978-3-7091-6139-5_23 12456068

[B87] Hyman B. Hoesen G. V. Damasio A. Barnes C. (1984). Alzheimer’s disease: cell-specific pathology isolates the hippocampal formation. *Science* 225 1168–1170. 10.1126/science.6474172 6474172

[B88] Hyman B. T. Van Hoesen G. W. Damasio A. R. (1990). Memory-related neural systems in Alzheimer’s disease: an anatomic study. *Neurology* 40 1721–1730. 2234428 10.1212/wnl.40.11.1721

[B89] Ibanez C. F. (2002). Jekyll-Hyde neurotrophins: the story of proNGF. *Trends Neurosci.* 25 284–286. 10.1016/s0166-2236(02)02169-0 12086739

[B90] Ibanez C. F. Simi A. (2012). p75 neurotrophin receptor signaling in nervous system injury and degeneration: paradox and opportunity. *Trends Neurosci.* 35 431–440. 10.1016/j.tins.2012.03.007 22503537

[B91] Iqbal K. Gong C. X. Liu F. (2013). Hyperphosphorylation-induced tau oligomers. *Front. Neurol.* 4:112. 10.3389/fneur.2013.00112 23966973 PMC3744035

[B92] Iulita M. F. Do Carmo S. Ower A. K. Fortress A. M. Flores Aguilar L. Hanna M. (2014). Nerve growth factor metabolic dysfunction in down’s syndrome brains. *Brain* 137(Pt 3), 860–872. 10.1093/brain/awt372 24519975 PMC3927704

[B93] James M. L. Belichenko N. P. Shuhendler A. J. Hoehne A. Andrews L. E. Condon C. (2017). [(18)F]GE-180 PET detects reduced microglia activation after LM11A-31 therapy in a mouse model of alzheimer’s disease. *Theranostics* 7 1422–1436. 10.7150/thno.17666 28529627 PMC5436503

[B94] Jang S. W. Okada M. Sayeed I. Xiao G. Stein D. Jin P. (2007). Gambogic amide, a selective agonist for TrkA receptor that possesses robust neurotrophic activity, prevents neuronal cell death. *Proc. Natl. Acad. Sci. U.S.A.* 104 16329–16334. 10.1073/pnas.0706662104 17911251 PMC2042206

[B95] Jette N. Cole M. S. Fahnestock M. (1994). NGF mRNA is not decreased in frontal cortex from Alzheimer’s disease patients. *Brain Res. Mol. Brain Res.* 25 242–250. 10.1016/0169-328x(94)90159-7 7808223

[B96] Johannsen P. (2006). Assessing therapeutic efficacy in a progressive disease: a study of donepezil in Alzheimer’s disease. *CNS Drugs* 20 311–325. 10.2165/00023210-200620040-00005 16599649

[B97] Johnson J. Pa J. Boxer A. Kramer J. Freeman K. Yaffe K. (2010). Baseline predictors of clinical progression among patients with dysexecutive mild cognitive impairment. *Dement. Geriatr. Cogn. Disord.* 30 344–351. 10.1159/000318836 20938178 PMC2975734

[B98] Jonhagen M. E. (2000). Nerve growth factor treatment in dementia. *Alzheimer Dis. Assoc. Disord.* 14(Suppl. 1), S31–S38. 10850728 10.1097/00002093-200000001-00006

[B99] Julien C. Tremblay C. Emond V. Lebbadi M. Salem N Jr. Bennett D. A. (2009). SIRT1 decrease parallels the accumulation of tau in Alzheimer disease. *J. Neuropathol. Exp. Neurol.* 68 48–58. 10.1097/NEN.0b013e3181922348 19104446 PMC2813570

[B100] Kaplan D. R. Miller F. D. (2004). Neurobiology: a move to sort life from death. *Nature* 427 798–799. 10.1038/427798a 14985746

[B101] Knowles J. Rajadas J. Nguyen T. Yang T. LeMieux M. Griend L. V. (2009). The p75 neurotrophin receptor promotes amyloid-β(1-42)-induced neuritic dystrophy in vitro and in vivo. *J. Neurosci.* 29 10627–10637. 10.1523/JNEUROSCI.0620-09.2009 19710315 PMC2771439

[B102] Knowles J. K. Simmons D. A. Nguyen T. V. Vander Griend L. Xie Y. Zhang H. (2013). Small molecule p75NTR ligand prevents cognitive deficits and neurite degeneration in an Alzheimer’s mouse model. *Neurobiol. Aging* 34 2052–2063. 10.1016/j.neurobiolaging.2013.02.015 23545424 PMC9035212

[B103] Kopeikina K. J. Carlson G. A. Pitstick R. Ludvigson A. E. Peters A. Luebke J. I. (2011). Tau accumulation causes mitochondrial distribution deficits in neurons in a mouse model of tauopathy and in human Alzheimer’s disease brain. *Am. J. Pathol.* 179 2071–2082. 10.1016/j.ajpath.2011.07.004 21854751 PMC3181340

[B104] Lalla R. Donmez G. (2013). The role of sirtuins in Alzheimer’s disease. *Front. Aging Neurosci.* 5:16 10.3389/fnagi.2013.00016 PMC3620486 23576985

[B105] Lasagna-Reeves C. A. Castillo-Carranza D. L. Sengupta U. Guerrero-Munoz M. J. Kiritoshi T. Neugebauer V. (2012). Alzheimer brain-derived tau oligomers propagate pathology from endogenous tau. *Sci. Rep.* 2:700. 10.1038/srep00700 23050084 PMC3463004

[B106] Lee R. Kermani P. Teng K. K. Hempstead B. L. (2001). Regulation of cell survival by secreted proneurotrophins. *Science* 294 1945–1948. 10.1126/science.1065057 11729324

[B107] Levi-Montalcini R. (2000). From Turin to Stockholm via St. Louis and Rio de Janeiro. *Science* 287:809. 10.1126/science.287.5454.809 10691554

[B108] Longo F. M. Yang T. Knowles J. K. Xie Y. Moore L. A. Massa S. M. (2007). Small molecule neurotrophin receptor ligands: novel strategies for targeting Alzheimer’s disease mechanisms. *Curr. Alzheimer Res.* 4 503–506. 10.2174/156720507783018316 18220511

[B109] Lorenzl S. Albers D. S. LeWitt P. A. Chirichigno J. W. Hilgenberg S. L. Cudkowicz M. E. (2003). Tissue inhibitors of matrix metalloproteinases are elevated in cerebrospinal fluid of neurodegenerative diseases. *J. Neurol. Sci.* 207 71–76. 10.1016/s0022-510x(02)00398-2 12614934

[B110] Lorenzl S. Buerger K. Hampel H. Beal M. F. (2008). Profiles of matrix metalloproteinases and their inhibitors in plasma of patients with dementia. *Int. Psychogeriatr.* 20 67–76. 10.1017/s1041610207005790 17697439

[B111] Luna-Munoz J. Chavez-Macias L. Garcia-Sierra F. Mena R. (2007). Earliest stages of tau conformational changes are related to the appearance of a sequence of specific phospho-dependent tau epitopes in Alzheimer’s disease. *J. Alzheimers Dis.* 12 365–375. 10.3233/jad-2007-12410 18198423

[B112] Maeda S. Sahara N. Saito Y. Murayama M. Yoshiike Y. Kim H. (2007). Granular tau oligomers as intermediates of tau filaments. *Biochemistry* 46 3856–3861. 10.1021/bi0613590 17338548

[B113] Mahady L. Nadeem M. Malek-Ahmadi M. Chen K. Perez S. Mufson E. (2018). Frontal cortex epigenetic dysregulation during the progression of alzheimer’s disease. *J. Alzheimer’s Dis.* 62 115–131. 10.3233/JAD-171032 29439356

[B114] Mahady L. Nadeem M. Malek-Ahmadi M. Chen K. Perez S. E. Mufson E. J. (2019). HDAC2 dysregulation in the nucleus basalis of meynert during the progression of Alzheimer’s disease. *Neuropathol. Appl. Neurobiol.* 45 380–397. 10.1111/nan.12518 30252960 PMC6433556

[B115] Mamidipudi V. Wooten M. W. (2002). Dual role for p75(NTR) signaling in survival and cell death: can intracellular mediators provide an explanation? *J. Neurosci. Res.* 68 373–384. 10.1002/jnr.10244 11992464

[B116] Mangialasche F. Solomon A. Winblad B. Mecocci P. Kivipelto M. (2010). Alzheimer’s disease: clinical trials and drug development. *Lancet Neurol.* 9 702–716. 10.1016/S1474-4422(10)70119-8 20610346

[B117] Mann D. M. Esiri M. M. (1989). The pattern of acquisition of plaques and tangles in the brains of patients under 50 years of age with down’s syndrome. *J. Neurol. Sci.* 89 169–179. 10.1016/0022-510x(89)90019-1 2522541

[B118] Markesbery W. R. (2010). Neuropathologic alterations in mild cognitive impairment: a review. *J. Alzheimers Dis.* 19 221–228. 10.3233/JAD-2010-1220 20061641 PMC2872776

[B119] Markesbery W. R. Schmitt F. A. Kryscio R. J. Davis D. G. Smith C. D. Wekstein D. R. (2006). Neuropathologic substrate of mild cognitive impairment. *Arch. Neurol.* 63 38–46. 10.1001/archneur.63.1.38 16401735

[B120] Massa S. M. Xie Y. Yang T. Harrington A. W. Kim M. L. Yoon S. O. (2006). Small, nonpeptide p75NTR ligands induce survival signaling and inhibit proNGF-induced death. *J. Neurosci.* 26 5288–5300. 10.1523/JNEUROSCI.3547-05.2006 16707781 PMC6675309

[B121] Mastroeni D. Grover A. Delvaux E. Whiteside C. Coleman P. D. Rogers J. (2010). Epigenetic changes in Alzheimer’s disease: decrements in DNA methylation. *Neurobiol. Aging* 31 2025–2037. 10.1016/j.neurobiolaging.2008.12.005 19117641 PMC2962691

[B122] Matyi J. Tschanz J. T. Rattinger G. B. Sanders C. Vernon E. K. Corcoran C. (2017). Sex differences in risk for alzheimer’s disease related to neurotrophin gene polymorphisms: the cache county memory study. *J. Gerontol. A Biol. Sci. Med. Sci.* 72 1607–1613. 10.1093/gerona/glx092 28498887 PMC5861928

[B123] McArthur J. C. Yiannoutsos C. Simpson D. M. Adornato B. T. Singer E. J. Hollander H. (2000). A phase II trial of nerve growth factor for sensory neuropathy associated with HIV infection. AIDS Clinical Trials Group Team 291. *Neurology* 54 1080–1088. 10.1212/wnl.54.5.1080 10720278

[B124] McKhann G. Drachman D. Folstein M. Katzman R. Price D. Stadlan E. M. (1984). Clinical diagnosis of Alzheimer’s disease: report of the NINCDS-ADRDA Work Group under the auspices of department of health and human services task force on alzheimer’s disease. *Neurology* 34 939–944. 6610841 10.1212/wnl.34.7.939

[B125] Mesulam M. Shaw P. Mash D. Weintraub S. (2004). Cholinergic nucleus basalis tauopathy emerges early in the aging-MCI-AD continuum. *Ann. Neurol.* 55 815–828. 10.1002/ana.20100 15174015

[B126] Mesulam M. M. Mufson E. J. Levey A. I. Wainer B. H. (1983). Cholinergic innervation of cortex by the basal forebrain: cytochemistry and cortical connections of the septal area, diagonal band nuclei, nucleus basalis (substantia innominata), and hypothalamus in the rhesus monkey. *J. Comp. Neurol.* 214 170–197. 10.1002/cne.902140206 6841683

[B127] Minnone G. Soligo M. Caiello I. Prencipe G. Manni L. Marafon D. P. (2017). ProNGF-p75NTR axis plays a proinflammatory role in inflamed joints: a novel pathogenic mechanism in chronic arthritis. *RMD Open* 3:e000441. 10.1136/rmdopen-2017-000441 28955492 PMC5604749

[B128] Mufson E. J. Binder L. Counts S. E. DeKosky S. T. de Toledo-Morrell L. Ginsberg S. D. (2012a). Mild cognitive impairment: pathology and mechanisms. *Acta Neuropathol.* 123 13–30. 10.1007/s00401-011-0884-1 22101321 PMC3282485

[B129] Mufson E. J. He B. Nadeem M. Perez S. Counts S. Leurgans S. (2012b). Hippocampal ProNGF signaling pathways and β-amyloid levels in mild cognitive impairment and alzheimer disease. *J. Neuropathol. Exp. Neurol.* 71 1018–1029. 10.1097/NEN.0b013e318272caab 23095849 PMC3481187

[B130] Mufson E. J. Malek-Ahmadi M. Perez S. Chen K. (2016a). Braak staging, plaque pathology and APOE status in elderly persons without cognitive impairment. *Neurobiol. Aging* 37 147–153. 10.1016/j.neurobiolaging.2015.10.012 26686670 PMC4687022

[B131] Mufson E. J. Malek-Ahmadi M. Snyder N. Ausdemore J. Chen K. Perez S. E. (2016b). Braak stage and trajectory of cognitive decline in noncognitively impaired elders. *Neurobiol. Aging* 43 101–110. 10.1016/j.neurobiolaging.2016.03.003 27255819 PMC4894536

[B132] Mufson E. J. Bothwell M. Kordower J. H. (1989). Loss of nerve growth factor receptor-containing neurons in Alzheimer’s disease: a quantitative analysis across subregions of the basal forebrain. *Exp. Neurol.* 105 221–232. 10.1016/0014-4886(89)90124-6 2548888

[B133] Mufson E. J. Counts S. E. Ginsberg S. D. (2002a). Gene expression profiles of cholinergic nucleus basalis neurons in Alzheimer’s disease. *Neurochem. Res.* 27 1035–1048. 12462403 10.1023/a:1020952704398

[B134] Mufson E. J. Ma S. Y. Dills J. Cochran E. J. Leurgans S. Wuu J. (2002b). Loss of basal forebrain P75(NTR) immunoreactivity in subjects with mild cognitive impairment and Alzheimer’s disease. *J. Comp. Neurol.* 443 136–153. 10.1002/cne.10122 11793352

[B135] Mufson E. J. Ginsberg S. D. Ikonomovic M. D. DeKosky S. T. (2003). Human cholinergic basal forebrain: chemoanatomy and neurologic dysfunction. *J. Chem. Neuroanat.* 26 233–242. 10.1016/s0891-0618(03)00068-1 14729126

[B136] Mufson E. J. Kordower J. H. (1992). Cortical neurons express nerve growth factor receptors in advanced age and Alzheimer disease. *Proc. Natl. Acad. Sci. U.S.A.* 89 569–573. 10.1073/pnas.89.2.569 1309947 PMC48280

[B137] Mufson E. J. Kordower J. H. (1999). “Nerve growth factor in Alzheimer’s disease,” in *Cerebral Cortex*, eds Peter A. A. Morrison J. H. (New York, NY: Kluwer Academic/Plenum Press), 681–731.

[B138] Mufson E. J. Ma S. J. Cochran E. J. Bennett D. A. Beckett L. A. Jaffar S. (2000). Loss of nucleus basalis neurons containing trkA immunoreactivity in individuals with mild cognitive impairment and early Alzheimer’s disease. *J. Comp. Neurol.* 427 19–30. 10.1002/1096-9861(20001106)427 11042589

[B2000] Mufson E. J. Mahady L. Waters D. Counts S. E. Perez S. E. DeKosky S. T. (2015).Hippocampal plasticity during the progression of Alzheimer’s disease. *Neuroscience* 309 51–67. 10.1016/j.neuroscience.2015.03.006 25772787 PMC4567973

[B139] Mufson E. J. Wuu J. Counts S. E. Nykjaer A. (2010). Preservation of cortical sortilin protein levels in MCI and Alzheimer’s disease. *Neurosci. Lett.* 47 1129–1133. 10.1016/j.neulet.2010.01.023 20085800 PMC2829104

[B140] Murase K. Nabeshima T. Robitaille Y. Quirion R. Ogawa M. Hayashi K. (1993). NGF level of is not decreased in the serum, brain-spinal fluid, hippocampus, or parietal cortex of individuals with Alzheimer’s disease. *Biochem. Biophys. Res. Commun.* 193 198–203. 10.1006/bbrc.1993.1609 8503908

[B141] Murphy M. Wilson Y. M. Vargas E. Munro K. M. Smith B. Huang A. (2015). Reduction of p75 neurotrophin receptor ameliorates the cognitive deficits in a model of Alzheimer’s disease. *Neurobiol. Aging* 36 740–752. 10.1016/j.neurobiolaging.2014.09.014 25443284

[B142] Murray S. S. Perez P. Lee R. Hempstead B. L. Chao M. V. (2004). A novel p75 neurotrophin receptor-related protein, NRH2, regulates nerve growth factor binding to the TrkA receptor. *J. Neurosci.* 24 2742–2749. 10.1523/JNEUROSCI.3960-03.2004 15028767 PMC6729530

[B143] Nabeshima T. Nitta A. Fuji K. Kameyama T. Hasegawa T. (1994). Oral administration of NGF synthesis stimulators recovers reduced brain NGF content in aged rats and cognitive dysfunction in basal-forebrain-lesioned rats. *Gerontology* 40(Suppl. 2), 46–56. 10.1159/000213627 7926866

[B144] Nair J. Klaassen A. L. Arato J. Vyssotski A. L. Harvey M. Rainer G. (2018). Basal forebrain contributes to default mode network regulation. *Proc. Natl. Acad. Sci. U.S.A.* 115 1352–1357. 10.1073/pnas.1712431115 29363595 PMC5819396

[B145] Narisawa-Saito M. Wakabayashi K. Tsuji S. Takahashi H. Nawa H. (1996). Regional specificity of alterations in NGF, BDNF and NT-3 levels in Alzheimer’s disease. *Neuroreport* 7 2925–2928. 10.1097/00001756-199611250-00024 9116211

[B146] Nguyen T. Shen L. Griend L. V. Quach L. Belichenko N. Saw N. (2014). Small molecule p75NTR ligands reduce pathological phosphorylation and misfolding of tau, inflammatory changes, cholinergic degeneration, and cognitive deficits in AβPP(L/S) transgenic mice. *J. Alzheimers Dis.* 42 459–483. 10.3233/jad-140036 24898660 PMC4278429

[B147] Nuydens R. Dispersyn G. de Jong M. van den Kieboom G. Borgers M. Geerts H. (1997). Aberrant tau phosphorylation and neurite retraction during NGF deprivation in PC12 cells. *Biochem. Biophys. Res. Commun.* 240 687–691. 10.1006/bbrc.1997.7721 9398627

[B148] Nyborg A. C. Ladd T. B. Zwizinski C. W. Lah J. J. Golde T. E. (2006). Sortilin, SorCS1b, and SorLA Vps10p sorting receptors, are novel gamma-secretase substrates. *Mol. Neurodegener.* 1:3. 16930450 10.1186/1750-1326-1-3 PMC1513133

[B149] Nykjaer A. Lee R. Teng K. K. Jansen P. Madsen P. Nielsen M. S. (2004). Sortilin is essential for proNGF-induced neuronal cell death. *Nature* 427 843–848. 10.1038/nature02319 14985763

[B150] Nykjaer A. Willnow T. Petersen C. (2005). p75NTR–live or let die. *Curr. Opin. Neurobiol.* 15 49–57. 10.1016/j.conb.2005.01.004 15721744

[B151] Pandey U. Nie Z. Batlevi Y. Mccray B. Ritson G. Nedelsky N. (2007). HDAC6 rescues neurodegeneration and provides an essential link between autophagy and the UPS. *Nature* 447 859–863. 17568747 10.1038/nature05853

[B152] Patterson K. R. Remmers C. Fu Y. Brooker S. Kanaan N. M. Vana L. (2011). Characterization of prefibrillar Tau oligomers in vitro and in Alzheimer disease. *J. Biol. Chem.* 286 23063–23076. 10.1074/jbc.M111.237974 21550980 PMC3123074

[B153] Pedraza C. E. Podlesniy P. Vidal N. Arevalo J. C. Lee R. Hempstead B. (2005). Pro-NGF isolated from the human brain affected by Alzheimer’s disease induces neuronal apoptosis mediated by p75NTR. *Am. J. Pathol.* 166 533–543. 10.1016/S0002-9440(10)62275-4 15681836 PMC1602327

[B154] Peng S. Wuu J. Mufson E. J. Fahnestock M. (2004). Increased proNGF levels in subjects with mild cognitive impairment and mild alzheimer’s disease. *J. Neuropathol. Exp. Neurol.* 63 641–649. 10.1093/jnen/63.6.641 15217092

[B155] Perez S. E. He B. Nadeem M. Wuu J. Scheff S. W. Abrahamson E. E. (2015). Resilience of precuneus neurotrophic signaling pathways despite amyloid pathology in prodromal Alzheimer’s disease. *Biol. Psychiatry* 77 693–703. 10.1016/j.biopsych.2013.12.016 24529280 PMC4096429

[B156] Petersen R. (2004). Mild cognitive impairment as a diagnostic entity. *J. Intern. Med.* 256 183–194. 10.1111/j.1365-2796.2004.01388.x 15324362

[B157] Petersen R. C. Smith G. E. Waring S. C. Ivnik R. J. Tangalos E. G. Kokmen E. (1999). Mild cognitive impairment: clinical characterization and outcome. *Arch. Neurol.* 56 303–308. 10190820 10.1001/archneur.56.3.303

[B158] Podlesniy P. Kichev A. Pedraza C. Saurat J. Encinas M. Perez B. (2006). Pro-NGF from Alzheimer’s disease and normal human brain displays distinctive abilities to induce processing and nuclear translocation of intracellular domain of p75NTR and apoptosis. *Am. J. Pathol.* 169 119–131. 10.2353/ajpath.2006.050787 16816366 PMC1698760

[B159] Poirier J. Delisle M.-C. Quirion R. Aubert I. Farlow M. Lahiri D. (1995). Apolipoprotein E4 allele as a predictor of cholinergic deficits and treatment outcome in alzheimer’s disease. *Proc. Natl. Acad. Sci. U.S.A.* 92 12260–12264. 10.1073/pnas.92.26.12260 8618881 PMC40336

[B160] Price J. L. Morris J. C. (1999). Tangles and plaques in nondemented aging and “preclinical” Alzheimer’s disease. *Ann. Neurol.* 45 358–368. 10.1002/1531-8249(199903)45 10072051

[B161] Reitz C. Tosto G. Vardarajan B. Rogaeva E. Ghani M. Rogers R. S. (2013). Independent and epistatic effects of variants in VPS10-d receptors on Alzheimer disease risk and processing of the amyloid precursor protein (APP). *Transl. Psychiatry* 3:e256. 10.1038/tp.2013.13 23673467 PMC3669917

[B162] Reynolds C. H. Nebreda A. R. Gibb G. M. Utton M. A. Anderton B. H. (1997). Reactivating kinase/p38 phosphorylates tau protein in vitro. *J. Neurochem.* 69 191–198. 10.1046/j.1471-4159.1997.69010191.x 9202310

[B163] Rosa E. Mahendram S. Ke Y. D. Ittner L. M. Ginsberg S. D. Fahnestock M. (2016). Tau downregulates BDNF expression in animal and cellular models of Alzheimer’s disease. *Neurobiol. Aging* 48 135–142. 10.1016/j.neurobiolaging.2016.08.020 27676333 PMC5159317

[B164] Roux P. P. Barker P. A. (2002). Neurotrophin signaling through the p75 neurotrophin receptor. *Prog. Neurobiol.* 67 203–233. 10.1016/s0301-0082(02)00016-3 12169297

[B165] Ryu J. C. Tooke K. Malley S. E. Soulas A. Weiss T. Ganesh N. (2018). Role of proNGF/p75 signaling in bladder dysfunction after spinal cord injury. *J. Clin. Invest.* 128 1772–1786. 10.1172/JCI97837 29584618 PMC5919823

[B166] Sadot E. Heicklen-Klein A. Barg J. Lazarovici P. Ginzburg I. (1996). Identification of a tau promoter region mediating tissue-specific-regulated expression in PC12 cells. *J. Mol. Biol.* 256 805–812. 10.1006/jmbi.1996.0126 8601831

[B167] Sahara N. DeTure M. Ren Y. Ebrahim A. S. Kang D. Knight J. (2013). Characteristics of TBS-extractable hyperphosphorylated tau species: aggregation intermediates in rTg4510 mouse brain. *J. Alzheimers Dis.* 33 249–263. 10.3233/JAD-2012-121093 22941973 PMC3514650

[B168] Sassin I. Schultz C. Thal D. R. Rub U. Arai K. Braak E. (2000). Evolution of Alzheimer’s disease-related cytoskeletal changes in the basal nucleus of Meynert. *Acta Neuropathol.* 100 259–269. 10.1007/s004019900178 10965795

[B169] Schindowski K. Belarbi K. Buee L. (2008). Neurotrophic factors in Alzheimer’s disease: role of axonal transport. *Genes Brain Behav.* 7(Suppl. 1), 43–56. 10.1111/j.1601-183X.2007.00378.x 18184369 PMC2228393

[B170] Schmitz T. W. Nathan Spreng R. (2016). Basal forebrain degeneration precedes and predicts the cortical spread of Alzheimer’s pathology. *Nat. Commun.* 7:13249. 10.1038/ncomms13249 27811848 PMC5097157

[B171] Schwab M. E. Otten U. Agid Y. Thoenen H. (1979). Nerve growth factor (NGF) in the rat CNS: absence of specific retrograde axonal transport and tyrosine hydroxylase induction in locus coeruleus and substantia nigra. *Brain Res.* 168 473–483. 10.1016/0006-8993(79)90303-2 86378

[B172] Scott S. A. Mufson E. Weingartner J. Skau K. Crutcher K. (1995). Nerve growth factor in alzheimer’s disease: increased levels throughout the brain coupled with declines in nucleus basalis. *J. Neurosci.* 15 6213–6221. 10.1523/jneurosci.15-09-06213.1995 7666203 PMC6577665

[B173] Sendera T. Ma S. Jaffar S. Kozlowski P. Kordower J. Mawal Y. (2000). Reduction in TrkA-immunoreactive neurons is not associated with an overexpression of galaninergic fibers within the nucleus basalis in down’s syndrome. *J. Neurochem.* 74 1185–1196. 10.1046/j.1471-4159.2000.741185.x 10693951

[B174] Shelton S. B. Johnson G. V. (2001). Tau and HMW tau phosphorylation and compartmentalization in apoptotic neuronal PC12 cells. *J. Neurosci. Res.* 66 203–213. 10.1002/jnr.1212 11592115

[B175] Shen L. L. Manucat-Tan N. B. Gao S. H. Li W. W. Zeng F. Zhu C. (2018). The ProNGF/p75NTR pathway induces tau pathology and is a therapeutic target for FTLD-tau. *Mol. Psychiatry* 23 1813–1824. 10.1038/s41380-018-0071-z 29867188

[B176] Shen X. Chen J. Li J. Kofler J. Herrup K. (2016). Neurons in vulnerable regions of the alzheimer’s disease brain display reduced ATM signaling. *eNeuro* 3:ENEURO.0124-15.2016. 10.1523/ENEURO.0124-15.2016 27022623 PMC4770009

[B177] Shoji M. Golde T. E. Ghiso J. Cheung T. T. Estus S. Shaffer L. M. (1992). Production of the alzheimer amyloid beta protein by normal proteolytic processing. *Science* 258 126–129. 10.1126/science.1439760 1439760

[B178] Simmons D. A. Knowles J. K. Belichenko N. P. Banerjee G. Finkle C. Massa S. M. (2014). A small molecule p75NTR ligand, LM11A-31, reverses cholinergic neurite dystrophy in Alzheimer’s disease mouse models with mid- to late-stage disease progression. *PLoS One* 9:e102136. 10.1371/journal.pone.0102136 25153701 PMC4143160

[B179] Skaper S. D. (2008). The biology of neurotrophins, signalling pathways, and functional peptide mimetics of neurotrophins and their receptors. *CNS Neurol. Disord. Drug Targets* 7 46–62. 10.2174/187152708783885174 18289031

[B180] Smith D. E. Roberts J. Gage F. H. Tuszynski M. H. (1999). Age-associated neuronal atrophy occurs in the primate brain and is reversible by growth factor gene therapy. *Proc. Natl. Acad. Sci. U.S.A.* 96 10893–10898. 10.1073/pnas.96.19.10893 10485922 PMC17979

[B181] Sotthibundhu A. Sykes A. Fox B. Underwood C. Thangnipon W. Coulson E. (2008). Beta-amyloid(1-42) induces neuronal death through the p75 neurotrophin receptor. *J. Neurosci.* 28 3941–3946. 10.1523/JNEUROSCI.0350-08.2008 18400893 PMC6670462

[B182] Sperling R. Mormino E. Johnson K. (2014). The evolution of preclinical Alzheimer’s disease: implications for prevention trials. *Neuron* 84 608–622. 10.1016/j.neuron.2014.10.038 25442939 PMC4285623

[B183] Sramek J. J. Cutler N. R. (1999). Recent developments in the drug treatment of Alzheimer’s disease. *Drugs Aging* 14 359–373. 10.2165/00002512-199914050-00004 10408736

[B184] Summers W. K. Majovski L. V. Marsh G. M. Tachiki K. Kling A. (1986). Oral tetrahydroaminoacridine in long-term treatment of senile dementia, Alzheimer type. *N. Engl. J. Med.* 315 1241–1245. 10.1056/NEJM198611133152001 2430180

[B185] Teng H. K. Teng K. K. Lee R. Wright S. Tevar S. Almeida R. D. (2005). ProBDNF induces neuronal apoptosis via activation of a receptor complex of p75NTR and sortilin. *J. Neurosci.* 25 5455–5463. 10.1523/jneurosci.5123-04.2005 15930396 PMC6724992

[B186] Teng K. K. Hempstead B. L. (2004). Neurotrophins and their receptors: signaling trios in complex biological systems. *Cell Mol. Life Sci.* 61 35–48. 10.1007/s00018-003-3099-3 14704852 PMC11138791

[B187] Tep C. Lim T. H. Ko P. O. Getahun S. Ryu J. C. Goettl V. M. (2013). Oral administration of a small molecule targeted to block proNGF binding to p75 promotes myelin sparing and functional recovery after spinal cord injury. *J. Neurosci.* 33 397–410. 10.1523/JNEUROSCI.0399-12.2013 23303920 PMC3710149

[B188] Thinakaran G. Koo E. H. (2008). Amyloid precursor protein trafficking, processing, and function. *J. Biol. Chem.* 283 29615–29619. 10.1074/jbc.R800019200 18650430 PMC2573065

[B189] Tiernan C. T. Ginsberg S. D. Guillozet-Bongaarts A. L. Ward S. M. He B. Kanaan N. M. (2016). Protein homeostasis gene dysregulation in pretangle bearing nucleus basalis neurons during the progression of Alzheimer’s disease. *Neurobiol. Aging* 42 80–90. 10.1016/j.neurobiolaging.2016.02.031 27143424 PMC4973891

[B190] Tiernan C. T. Ginsberg S. D. He B. Ward S. M. Guillozet-Bongaarts A. L. Kanaan N. M. (2018a). Pretangle pathology within cholinergic nucleus basalis neurons coincides with neurotrophic and neurotransmitter receptor gene dysregulation during the progression of Alzheimer’s disease. *Neurobiol. Dis.* 117 125–136. 10.1016/j.nbd.2018.05.021 29859871 PMC6278831

[B191] Tiernan C. T. Mufson E. J. Kanaan N. M. Counts S. E. (2018b). Tau oligomer pathology in nucleus basalis neurons during the progression of Alzheimer’s disease. *J. Neuropathol. Exp. Neurol.* 77 246–359. 10.1016/j.nbd.2018.05.021 29378005 PMC6251641

[B192] Trojanowski J. Q. Schmidt M. L. Shin R. W. Bramblett G. T. Rao D. Lee V. M. (1993). Altered tau and neurofilament proteins in neuro-degenerative diseases: diagnostic implications for Alzheimer’s disease and Lewy body dementias. *Brain Pathol.* 3 45–54. 10.1111/j.1750-3639.1993.tb00725.x 8269083

[B193] Troy C. M. Friedman J. E. Friedman W. J. (2002). Mechanisms of p75-mediated death of hippocampal neurons. Role of caspases. *J. Biol. Chem.* 277 34295–34302. 10.1074/jbc.M205167200 12097334

[B194] Tuszynski M. Blesch A. (2004). Nerve growth factor: from animal models of cholinergic neuronal degeneration to gene therapy in Alzheimer’s disease. *Prog. Brain Res.* 146 441–449. 14699979 10.1016/s0079-6123(03)46028-7

[B195] Tuszynski M. H. Hs U. Amaral D. G. Gage F. H. (1990). Nerve growth factor infusion in the primate brain reduces lesion-induced cholinergic neuronal degeneration. *J. Neurosci.* 10 3604–3614. 10.1523/jneurosci.10-11-03604.1990 2230949 PMC6570113

[B196] Tuszynski M. H. Roberts J. Senut M. C. Hs U. Gage F. H. (1996). Gene therapy in the adult primate brain: intraparenchymal grafts of cells genetically modified to produce nerve growth factor prevent cholinergic neuronal degeneration. *Gene Ther.* 3 305–314. 8732162

[B197] Tuszynski M. H. Sang H. Yoshida K. Gage F. H. (1991). Recombinant human nerve growth factor infusions prevent cholinergic neuronal degeneration in the adult primate brain. *Ann. Neurol.* 30 625–636. 10.1002/ana.410300502 1763889

[B198] Tuszynski M. H. Thal L. Pay M. Salmon D. P. Hs U. Bakay R. (2005). A phase 1 clinical trial of nerve growth factor gene therapy for Alzheimer disease. *Nat. Med.* 11 551–555. 10.1038/nm1239 15852017

[B199] Tuszynski M. H. Yang J. H. Barba D. Hs U. Bakay R. A. Pay M. M. (2015). Nerve growth factor gene therapy: activation of neuronal responses in alzheimer disease. *JAMA Neurol.* 72 1139–1147. 10.1001/jamaneurol.2015.1807 26302439 PMC4944824

[B200] Ulrich E. Duwel A. Kauffmann-Zeh A. Gilbert C. Lyon D. Rudkin B. (1998). Specific TrkA survival signals interfere with different apoptotic pathways. *Oncogene* 16 825–832. 10.1038/sj.onc.1201842 9484773

[B201] Vana L. Kanaan N. M. Ugwu I. C. Wuu J. Mufson E. J. Binder L. I. (2011). Progression of tau pathology in cholinergic Basal forebrain neurons in mild cognitive impairment and Alzheimer’s disease. *Am. J. Pathol.* 179 2533–2550. 10.1016/j.ajpath.2011.07.044 21945902 PMC3204017

[B202] Volmar C. H. Claes W. (2015). Histone deacetylases (HDACs) and brain function. *Neuroepigenetics* 1 20–27. 10.1016/j.nepig.2014.10.002

[B203] Ward S. M. Himmelstein D. S. Lancia J. K. Fu Y. Patterson K. R. Binder L. I. (2013). TOC1: characterization of a selective oligomeric tau antibody. *J. Alzheimers Dis.* 37 593–602. 10.3233/JAD-131235 23979027 PMC4791958

[B204] Williams L. R. Varon S. Peterson G. M. Wictorin K. Fischer W. Bjorklund A. (1986). Continuous infusion of nerve growth factor prevents basal forebrain neuronal death after fimbria fornix transection. *Proc. Natl. Acad. Sci. U.S.A.* 83 9231–9235. 10.1073/pnas.83.23.9231 3466184 PMC387109

[B205] Wimo A. (2007). Clinical and economic outcomes–friend or foe? *Int. Psychogeriatr.* 19 497–507. 10.1017/s1041610207004930 17346367

[B206] Wimo A. Guerchet M. Ali G. C. Wu Y. T. Prina A. M. Winblad B. (2017). The worldwide costs of dementia 2015 and comparisons with 2010. *Alzheimers Dement.* 13 1–7. 10.1016/j.jalz.2016.07.150 27583652 PMC5232417

[B207] Wu C. K. Thal L. Pizzo D. Hansen L. Masliah E. Geula C. (2005). Apoptotic signals within the basal forebrain cholinergic neurons in Alzheimer’s disease. *Exp. Neurol.* 195 484–496. 10.1016/j.expneurol.2005.06.020 16085017

[B208] Xu K. Dai X. L. Huang H. C. Jiang Z. F. (2011). Targeting HDACs: a promising therapy for Alzheimer’s disease. *Oxid. Med. Cell Longev.* 2011:143269. 10.1155/2011/143269 21941604 PMC3177096

[B209] Yang T. Knowles J. Lu Q. Zhang H. Arancio O. Moore L. (2008). Small molecule, non-peptide p75NTR ligands inhibit aβ-induced neurodegeneration and synaptic impairment. *PLoS One* 3: e3604. 10.1371/journal.pone.0003604 18978948 PMC2575383

[B210] Yoon S. O. Casaccia-Bonnefil P. Carter B. Chao M. V. (1998). Competitive signaling between TrkA and p75 nerve growth factor receptors determines cell survival. *J. Neurosci.* 18 3273–3281. 10.1523/jneurosci.18-09-03273.1998 9547236 PMC6792655

[B211] Yoshiyama Y. Lee V. M. Trojanowski J. Q. (2013). Therapeutic strategies for tau mediated neurodegeneration. *J. Neurol. Neurosurg. Psychiatry* 84 784–795. 10.1136/jnnp-2012-303144 23085937 PMC3912572

[B212] Zhu X. Castellani R. Takeda A. Nunomura A. Atwood C. Perry G. (2001). Differential activation of neuronal ERK, JNK/SAPK and p38 in Alzheimer disease: the “two hit” hypothesis. *Mech. Ageing Dev.* 123 39–46. 10.1016/s0047-6374(01)00342-6 11640950

[B213] Zucker S. Hymowitz M. Conner C. Zarrabi H. M. Hurewitz A. N. Matrisian L. (1999). Measurement of matrix metalloproteinases and tissue inhibitors of metalloproteinases in blood and tissues. clinical and experimental applications. *Ann. N.Y. Acad. Sci.* 878 212–227. 10.1111/j.1749-6632.1999.tb07687.x 10415733

